# Hybrid ConvNeXtV2–ViT Architecture with Ontology-Driven Explainability and Out-of-Distribution Awareness for Transparent Chest X-Ray Diagnosis

**DOI:** 10.3390/diagnostics16020294

**Published:** 2026-01-16

**Authors:** Naif Almughamisi, Gibrael Abosamra, Adnan Albar, Mostafa Saleh

**Affiliations:** Department of Information Systems, Faculty of Computing and Information Technology, King Abdulaziz University, Jeddah 21589, Saudi Arabia; gabosamra@kau.edu.sa (G.A.); ambar@kau.edu.sa (A.A.); msherbini@kau.edu.sa (M.S.)

**Keywords:** hybrid CNN–ViT, interpretable model, grad-CAM, neuro-symbolic reasoning, thoracic diseases, out-of-distribution detection

## Abstract

**Background:** Chest X-ray (CXR) is widely used for the assessment of thoracic diseases, yet automated multi-label interpretation remains challenging due to subtle visual patterns, overlapping anatomical structures, and frequent co-occurrence of abnormalities. While recent deep learning models have shown strong performance, limitations in interpretability, anatomical awareness, and robustness continue to hinder their clinical adoption. **Methods:** The proposed framework employs a hybrid ConvNeXtV2–Vision Transformer (ViT) architecture that combines convolutional feature extraction for capturing fine-grained local patterns with transformer-based global reasoning to model long-range contextual dependencies. The model is trained exclusively using image-level annotations. In addition to classification, three complementary post hoc components are integrated to enhance model trust and interpretability. A segmentation-aware Gradient-weighted class activation mapping (Grad-CAM) module leverages CheXmask lung and heart segmentations to highlight anatomically relevant regions and quantify predictive evidence inside and outside the lungs. An ontology-driven neuro-symbolic reasoning layer translates Grad-CAM activations into structured, rule-based explanations aligned with clinical concepts such as “basal effusion” and “enlarged cardiac silhouette”. Furthermore, a lightweight out-of-distribution (OOD) detection module based on confidence scores, energy scores, and Mahalanobis distance scores is employed to identify inputs that deviate from the training distribution. **Results:** On the VinBigData test set, the model achieved a macro-AUROC of 0.9525 and a Micro AUROC of 0.9777 when trained solely with image-level annotations. External evaluation further demonstrated strong generalisation, yielding macro-AUROC scores of 0.9106 on NIH ChestXray14 and 0.8487 on CheXpert (frontal views). Both Grad-CAM visualisations and ontology-based reasoning remained coherent on unseen data, while the OOD module successfully flagged non-thoracic images. **Conclusions:** Overall, the proposed approach demonstrates that hybrid convolutional neural network (CNN)–vision transformer (ViT) architectures, combined with anatomy-aware explainability and symbolic reasoning, can support automated chest X-ray diagnosis in a manner that is accurate, transparent, and safety-aware.

## 1. Introduction

Chronic obstructive pulmonary disease (COPD) and interstitial lung disease, along with other forms of lung disorders such as pneumonia and pleural disorders, are among the most prevalent causes of morbidity and mortality worldwide. Chronic respiratory diseases are estimated to cause over 4 million deaths annually. Therefore, chronic respiratory diseases account for a significant proportion of global noncommunicable disease mortality [1,2]. Lung infections such as pneumonia and tuberculosis, together with COPD and lung cancer, are collectively responsible for approximately one in six deaths worldwide. Therefore, early identification and accurate monitoring of these conditions are crucial for improving outcomes and reducing the healthcare burden.

Chest X-ray (CXR) remains the most widely used and cost-effective imaging examination for initial assessment of thoracic disease. It is routinely used across inpatient and outpatient settings for screening, triage, and follow-up, supporting the detection of a broad range of abnormalities including pulmonary opacities, lung nodules, pleural disease, and cardiac enlargement [3]. However, CXR interpretation is inherently challenging because projections are two-dimensional, anatomical structures overlap, and image quality varies across scanners and acquisition protocols. Inter-observer variability among radiologists is also well documented, particularly for subtle findings such as early interstitial disease, mild cardiomegaly, and small pneumothorax [4].

Over the last decade, deep learning has been increasingly applied to large annotated CXR datasets. Convolutional neural networks (CNNs) have demonstrated strong performance for detecting individual pathologies [5]; however, their predominantly local receptive fields can limit the modelling of large-scale patterns and the relationships among co-occurring findings.

Although Chest X-rays are still by far the least expensive and most commonly used radiographic examination utilised at the initial stage of disease assessment of the thoracic cavity, they are also employed throughout patient care in all locations of healthcare (inpatient and outpatient), as well as a tool for screening, triage, and follow-up assessments for the detection of a variety of pulmonary abnormalities, such as, but not limited to, pulmonary opacities, lung nodules, pleural abnormalities, and cardiac enlargement [3]. However, interpreting CXRs is challenging because projections are two-dimensional, different disease patterns may overlap, and image quality can vary substantially across scanners and acquisition protocols. Inter-observer variability among radiologists is well documented, especially for subtle findings such as early interstitial disease, mild cardiomegaly, and small pneumothorax [4].

Over the last 10 years, deep learning has increasingly been applied to large, annotated collections of chest X-rays. CNNs are highly effective at identifying single pathologies from chest radiographs [5]; however, their limited receptive fields and inductive bias can inhibit their ability to detect larger-scale patterns or the interrelationships among findings. Recently, ViTs and a variety of hybrid CNN-ViT models have been developed to incorporate both local and spatial texture descriptors and global self-attention mechanisms to improve performance on a variety of CXR benchmarks [6,7,8]. These architectures have demonstrated that combining convolutional backbones with transformer-based encoders can boost the detection of pulmonary abnormalities while maintaining computational efficiency.

Although there have been significant advancements in radiology, several challenges remain unresolved. The first challenge is that multi-label prediction is challenging because multiple abnormalities can occur simultaneously in an image [9]. Additionally, the imbalance in label distribution across a single radiograph is high; common issues (lung opacity, cardiomegaly) comprise the majority of the training data, whereas uncommon issues (calcification, lesions) are found in fewer than 10% of radiographs [10]. The second major challenge is that most current models for classifying chest X-rays operate as a “black box” that produces only a probability score and does not provide explicit evidence for the classification. Saliency techniques (such as Grad-CAM) are commonly employed to create visual explanations of classification decisions; however, in many cases, the interpretations of saliency maps are qualitative rather than quantitative and thus cannot be integrated into a structured reasoning process [11]. Many studies still rely solely on qualitative inspection, without integrating structured reasoning or formal clinical knowledge [12]. Third, OOD detection is another critical requirement for clinical deployment. In medical imaging, OOD detection poses particular challenges owing to the wide range of imaging protocols, varied patient demographics, and diverse disease manifestations [13].

To address these limitations, there is a growing interest in combining deep neural networks with neuro-symbolic AI and ontological reasoning [14]. In such approaches, probabilistic predictions from CNN or transformer backbones are integrated with explicit knowledge graphs or ontologies that encode the relationships between findings, anatomical regions, and higher-level diagnoses. Clinical concepts such as “cardiomegaly, ” “pleural effusion, ” or “lung opacity” can be represented as nodes in an ontology, together with logical rules that define composite conditions and mutual exclusions. Large, standardised terminologies such as RadLex and SNOMED CT provide a rich vocabulary and hierarchical structure for radiological and clinical concepts, making them natural foundations for ontology-driven decision support. By aligning pixel-level evidence with these structured clinical rules, neuro-symbolic systems aim to produce more transparent and clinically meaningful outputs, such as textual explanations, consistency checks, and composite diagnoses, for example, heart failure as cardiomegaly plus pleural effusion rather than isolated labels. In the context of chest X-ray analysis, ontology-driven reasoning offers a natural way to move beyond flat probability vectors toward interpretable, knowledge-grounded diagnostic support [15].

In this study, we propose an enhanced hybrid architecture that combines ConvNeXtV2-Base and ViT-B/16 for multi-label classification of pulmonary diseases using the VinBigData chest X-ray abnormalities detection dataset, formulated as a 15-label classification problem (14 thoracic abnormalities plus a no-finding class) [16]. We additionally used VinDr-CXR-derived CheXmask segmentations to obtain lung and heart anatomical masks, enabling an anatomy-aware analysis of the model’s predictions [17]. The framework was designed with three key objectives:

- High-accuracy multi-label classification: To achieve robust prediction across 15 labels using a modern CNN–ViT hybrid trained with mixup augmentation, class-wise threshold optimisation, warm-up, cosine learning-rate scheduling, mixed-precision training, and test-time augmentation.
- Anatomy-aware explainability for multi-label outputs: To provide class-specific, anatomy-aware explanations by coupling Grad-CAM with lung and heart segmentation masks derived from CheXmask (VinDr-CXR), allowing the spatial focus of each predicted label to be quantified relative to the clinically relevant anatomical regions.
- Ontology-driven neuro-symbolic reasoning over multi-label predictions: A CXR-specific clinical decision support system ontology (CXR-CDSS) encodes VinBig-related findings and composite disease concepts, and model outputs together with Grad–CAM–based spatial evidence are mapped into this ontology so that an automated reasoner can derive higher-level case types and generate radiology-style textual explanations based on combinations of predicted labels.

Additionally, logit-based energy and maximum probability scores are incorporated as OOD indicators to flag suspicious inputs before generating automated reasoning panels. On the 20% hold-out test set, the proposed framework achieved a macro-AUROC of 0.9525, with strong performance for key clinical findings such as cardiomegaly, pleural effusion, and pulmonary fibrosis, while producing intuitive Grad-CAM overlays and ontology-supported diagnostic narratives.

### 1.1. Main Contributions

The main contributions of this study are summarised as follows:

- Multi-label hybrid architecture. A ConvNeXtV2–ViT hybrid model is designed specifically for the VinBigData chest X-ray abnormality detection task, formulated as a 15-label multi-label problem (14 abnormalities plus a no-finding class). The architecture is trained with mixup augmentation, class-wise threshold optimisation, and a modern training scheme that includes warm-up, cosine learning-rate scheduling, and mixed-precision training.
- Anatomy-aware multi-label explainability (post hoc). Grad-CAM explanations are integrated with post hoc anatomical masks (lungs and heart) from CheXmask to generate label-specific, anatomy-aware visual explanations. This enables quantitative analysis of whether activations fall within clinically relevant thoracic regions and how attention is distributed across anatomical subregions.
- Ontology-based neuro-symbolic reasoning for multi-label predictions. A CXR clinical decision-support ontology (CXR-CDSS) is implemented in OWL to encode VinBigData findings, anatomical structures, and composite disease concepts. Grad-CAM-derived spatial evidence is converted into fuzzy predicates and combined with predicted probabilities via rule-based reasoning to produce support scores and short diagnostic narratives for multi-label cases.
- Comprehensive evaluation beyond black-box classification. Strong multi-label detection performance is reported on the held-out VinBigData test split (macro/micro AUROC and fixed-threshold precision/F1). In addition, qualitative and quantitative analyses demonstrate that the explainability and ontology modules provide structured, knowledge-grounded interpretations that complement standard classifier outputs.
- Improved safety via post hoc OOD detection with quantitative benchmarking. A post hoc OOD layer is attached to the trained hybrid model using complementary scoring functions (confidence/entropy, energy, and Mahalanobis distance in fused feature space). A quantitative ID vs. OOD benchmark (VinBigData vs. non-chest radiographs) is reported using AUROC and operating-point metrics including ID-FPR and OOD-TPR, supporting robustness to clearly mismatched inputs.
- Reproducibility. Controlled random seeds, a detailed hyperparameter table, and an explicit evaluation protocol (split files, threshold fitting, and OOD calibration) are documented to improve reproducibility and facilitate re-use by other researchers.

The remainder of this paper is organised as follows. Section 2 describes the materials and methods, including the VinBigData dataset, the multi-label problem formulation, class-imbalance handling, the enhanced ConvNeXtV2–ViT hybrid architecture, Grad-CAM with CheXmask segmentations, OOD scoring, and the proposed ontology-driven reasoning pipeline. Section 3 presents the experimental setup and performance of the enhanced hybrid model, together with qualitative examples of anatomy-aware explanations and neuro-symbolic diagnostic reasoning. Section 4 discusses the results in the context of existing literature, highlighting the clinical implications, strengths, and limitations of the proposed framework. Finally, Section 5 summarises the study’s main conclusions.

### 1.2. Related Work

Advances in Automated Chest X-Ray Analysis are ongoing as researchers pursue greater levels of accuracy, robustness, and generalisability. Hybrid Architectures combining convolutional neural networks with transformer-based components have demonstrated substantial improvements in Multi-Label Disease Detection; however, these models still face considerable limitations, particularly in computational efficiency, susceptibility to overfitting, explicit modelling of clinical knowledge, out-of-distribution behaviour, and the provision of structured explanations.

Ait Nasser et al. [18] reviewed deep learning techniques for analysing chest X-rays and found that current CNN architectures (DenseNet and EfficientNet) have achieved high accuracy in detecting multiple diseases from a single image, reaching 0.90 on large public datasets. However, most surveyed approaches still treat the prediction vector as a flat set of labels, without explicitly reasoning about dependencies or relationships among abnormalities.

Ashraf et al. [8] introduced SynthEnsemble, a fusion of CNNs, vision transformers, and hybrid models for multi-label chest X-ray classification on the ChestX-ray14 dataset, where the best single backbone (CoAtNet) achieved high AUROC, and an ensemble of CNN–ViT models further improved multi-label performance. The study highlighted that hybrid architectures can better capture both local textures and global contexts than pure CNNs, but at the cost of increased computational complexity and training time. Yulvina et al. [19] presented a hybrid vision transformer and CNN model for multiclass and multi-label classification of tuberculosis anomalies on chest X-ray, combining convolutional layers with self-attention to handle complex patterns and using focal loss and class weighting to mitigate label imbalance. Their results confirmed that hybrid CNN–ViT designs offer benefits for multi-label chest imaging. However, the study focused on a smaller dataset and did not consider explainability beyond basic visualisation.

Fu et al. [20] proposed LungMaxViT, an explainable hybrid transformer that combines improved convolutional blocks with MaxViT modules for multiclass lung disease classification on the COVID-19 and ChestX-ray14 datasets. The model uses image enhancement (e.g., CLAHE and denoising) and extensive data augmentation, and Grad-CAM is employed to generate visual explanations for the predicted classes. LungMaxViT achieved very high AUC and accuracy, demonstrating the potential of transformer-enhanced backbones for thoracic imaging. However, the task is primarily framed as multiclass rather than fully multi-label, and the framework does not incorporate explicit OOD detection or ontology-based reasoning about combinations of findings.

Regarding the VinBigData chest X-ray abnormalities detection dataset, most published studies have treated it primarily as an object-detection problem. Nawaz et al. [21] presented one of the few fully documented deep learning pipelines that explicitly target the VinBigData chest X-ray abnormalities detection dataset. Their study employed a multi-level YOLOv5-based framework to localise and classify 14 VinBigData thoracic abnormalities. The authors used the official Kaggle training data, applied standard resizing and normalisation, and relied on transfer learning with YOLOv5 to detect lesions such as aortic enlargement, atelectasis, calcification, cardiomegaly, consolidation, interstitial lung disease, infiltration, lung opacity, pleural effusion, pleural thickening, pneumothorax, nodule/mass, and “other lesion”.

Explainable AI (XAI) plays a central role in recent chest X-ray studies. The Grad-CAM method introduced by Selvaraju et al. [11] remains one of the most widely used techniques for generating class-discriminative heatmaps by backpropagating the gradients to the last convolutional layer. It has been adopted in numerous medical imaging studies to highlight the model’s attention. Kansal et al. [22] proposed a transformer-based Grad-CAM network for lung disease classification, combining transformer encoders with a Grad-CAM-style explanation module to provide localisation maps for predicted abnormalities. Their findings suggest that transformer-based models can offer improved diagnostic accuracy while supporting interpretable visual explanations. Nevertheless, in most of these studies, Grad-CAM was used qualitatively, without systematic quantification of the amount of attention within specific anatomical regions, such as the lungs or heart, and without coupling the visual evidence to a higher-level reasoning component.

Robustness to out-of-distribution inputs has been recognised as an essential requirement for the safe deployment of deep models in clinical practice. Liu et al. [23] proposed an energy-based framework for OOD detection, showing that an energy score computed from a classifier’s logits can more reliably distinguish in-distribution from OOD samples than the maximum softmax probability and can be used both as a post hoc score and as a training objective. This method has inspired subsequent applications in medical imaging, where energy scores and related logit-based measures are used to flag unexpected inputs before issuing high-confidence predictions. However, many chest X-ray classification pipelines either omit explicit OOD handling or rely only on simple softmax-based confidence thresholds, and very few integrate OOD scores with explainability and knowledge-based reasoning.

Parallel to advances in neural architectures, there has been a long-standing line of work on ontologies and knowledge-based decision support in radiology. Budovec et al. [24] presented an ontology-based diagnostic decision support system built on the Radiology Gamuts Ontology, demonstrating how causal relationships between imaging findings and differential diagnoses can be exploited to provide interactive diagnostic suggestions for radiologists. Jing et al. [25] reviewed the use of ontologies in clinical decision support system rules, showing that ontologies such as SNOMED CT and RadLex are increasingly used to organise medical knowledge, structure decision rules, and improve the maintainability of CDSS logic.

These studies illustrate the potential of ontologies to encode relationships between findings, anatomical regions, and diagnoses; however, they typically operate on structured clinical data or text (e.g., radiology reports) rather than directly on pixel-level evidence from deep neural networks. Overall, existing studies demonstrate substantial progress in multi-label chest X-ray classification, hybrid CNN–Transformer architectures, and XAI, as well as parallel advances in ontology-based clinical decision support and OOD detection. However, most approaches address these components separately. There is still a gap in frameworks that simultaneously (i) use a modern hybrid CNN–Transformer backbone for multi-label classification on VinBigData, (ii) provide anatomy-aware Grad-CAM explanations using lung and heart masks, (iii) incorporate energy-based and softmax-based scores for OOD detection, and (iv) map both probabilistic outputs and spatial evidence into an explicit chest imaging ontology for neuro-symbolic reasoning and radiology-style textual reporting. This study aims to bridge this gap by integrating all four aspects into a single ontology-driven hybrid pipeline. As summarised in Table 1, several recent studies have explored hybrid and ontology-based frameworks for chest X-ray interpretation.

## 2. Materials and Methods

This study uses a developed hybrid architecture that combines a ConvNeXtV2 convolutional backbone with a Vision Transformer (ViT-B/16) to classify chest radiographs into 15 categories (14 thoracic abnormalities and one “no finding” category). Experiments were conducted using the VinBigData Chest X-Ray Abnormalities Detection Dataset, released by the Hanoi Medical University Hospital, Hanoi, Vietnam, which provides expert-annotated bounding box annotations for clinically significant thoracic abnormalities. This dataset enables correlation between pixel-level evidence, image-level labels, and neuro-symbolic reasoning.

All experiments were implemented using Python (version 3.10) with the PyTorch deep learning framework (version 2.1). Model training and evaluation were performed using standard scientific computing libraries, including NumPy (version 1.26) and OpenCV (version 4.11).

The ConvNeXtV2 serves as the CNN branch to extract local features at high resolution, whereas the ViT branch captures long-range dependencies and global context; both branches are initialised from ImageNet-pretrained models to stabilise optimisation and enhance generalisation. In the CNN branch, a ConvNeXtV2-Base network (without its classification head) produced a deep feature map for every radiograph. A multi-axis attention (MXA) block is applied on top of this feature map to enhance the diagnostically salient channels and spatial locations, and global average pooling yields a compact CNN feature vector. In parallel, a ViT-Base/16 model with a 384 × 384 nput resolution processes the same pre-processed image and outputs a global class token embedding. These two feature vectors were concatenated and passed through a fully connected layer to produce 15 logits corresponding to the VinBigData label set.

To align the model focus with clinically relevant anatomy, the framework supports an optional anatomy-aware loss that penalises strong MXA activations outside the thoracic cavity using lung and heart masks derived from the CheXmask (VinDr-CXR) dataset. In the VinBigData experiments reported here, this penalty was disabled, and the model was trained purely with image-level supervision; CheXmask masks were used only for post hoc evaluation and interpretability

**Figure 1:**
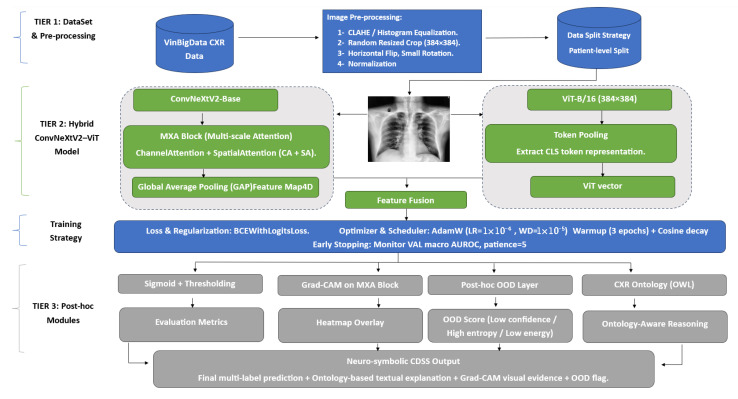
Overview of the proposed ConvNeXtV2–ViT framework for VinBigData. The model combines ConvNeXtV2-Base + MXA and ViT-B/16, and includes post hoc segmentation-aware Grad-CAM, ontology-based reasoning, and OOD detection.

On top of the trained hybrid network, we implemented three post hoc modules: (i) a segmentation-aware Grad-CAM pipeline that quantifies how well class-specific heatmaps align with anatomical masks, (ii) a neuro-symbolic layer that maps Grad-CAM evidence into fuzzy predicates and ontology-aligned rules, and (iii) a post hoc OOD detector based on confidence/entropy, energy, and Mahalanobis distance in feature space. Figure 1 illustrates the overall framework, including dual-branch feature extraction, Grad-CAM, OOD analyses, and ontology-based reasoning.

### 2.1. Dataset

This study used the public VinBigData Chest X-ray Abnormalities Detection dataset released for the VinBigData Challenge on Kaggle [26]. It includes 15,000 posteroanterior (PA) CXRs annotated by up to three radiologists with bounding boxes and labels for 14 thoracic abnormalities, plus a No finding label. The provided annotation file was organised at the bounding-box level, where each row corresponded to a single box, linked to an image identifier and a class label. To cast the task as multi-label classification, we converted the box-level annotations into image-level targets by aggregating all boxes with the same image identifier. Each radiograph is thus represented by a 15-dimensional binary target vector $y\in {\left\{0,1\right\}}^{15}$, where $y_{c}=1$ indicates the presence of the *c*-th finding and $y_{c}=0$ otherwise. The target vector covers the following classes:

- Aortic enlargement;
- Atelectasis;
- Calcification;
- Cardiomegaly;
- Consolidation;
- Interstitial lung disease;
- Infiltration;
- Lung opacity;
- Nodule/Mass;
- Other lesion;
- Pleural effusion;
- Pleural thickening;
- Pneumothorax;
- Pulmonary fibrosis;
- No finding.

Because multiple abnormalities may coexist within a single radiograph (e.g., cardiomegaly with pleural effusion) [27], VinBigData represents a genuine multi-label setting rather than a mutually exclusive multiclass problem. Across the 15,000 images, the average label cardinality is approximately 1.73 (range 1–10), which reflects substantial label co-occurrence. Images assigned only the No finding label and no abnormality labels were treated as normal cases; this corresponded to 10,606 radiographs (70.7%) of the dataset.

**Figure 2:**
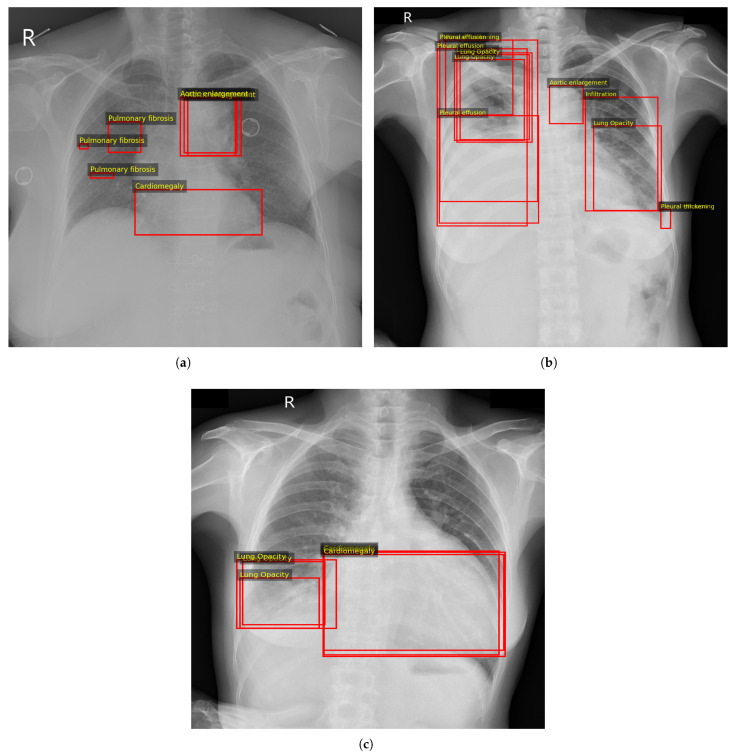
Examples of VinBigData radiologist bounding-box annotations overlaid with Grad-CAM visualisations for selected thoracic abnormalities. Red rectangular frames indicate expert-annotated regions of interest corresponding to abnormal findings. The letter “R” denotes the right side of the chest radiograph. Multi-label targets for the three examples are listed in Table 2. (**a**) Example 1. (**b**) Example 2. (**c**) Example 3.

Importantly, the bounding box coordinates were not used for supervision during training or for quantitative evaluations. The proposed model was trained solely using image-level multi-label targets derived from the aggregated annotations. The original bounding boxes were retained only for qualitative analysis, where they were overlaid on Grad-CAM visualisations to compare model attention with radiologist-marked lesion regions (Figure 2).

#### 2.1.1. Illustration of the Multi-Label Target Encoding

To make the multi-label encoding explicit, Table 2 lists the positive labels ($y_{c}=1$) for the three radiographs shown in Figure 2. Example (a) contains multiple concurrent findings, Example (b) illustrates a more complex multi-label case with several co-existing abnormalities, and Example (c) represents a simpler two-label case. All remaining classes not listed for a given example have target value 0.

#### 2.1.2. Class Distribution and Imbalance

Table 3 summarises the number of positive images per class in the VinBigData cohort, using the 70/10/20 train/validation/test split employed in this study (15,000 labelled radiographs). The label distribution was strongly skewed; common findings, such as aortic enlargement (20.45%), cardiomegaly (15.33%), and pleural thickening (13.21%), were observed in thousands of images. In contrast, rare abnormalities such as pneumothorax (0.64%) and atelectasis (1.24%) occurred in fewer than 200 radiographs. Because multiple findings may coexist within the same image, the learning problem is further complicated by label co-occurrence, which can amplify the effective imbalance and bias optimisation toward frequent labels if not explicitly addressed. The dataset has a 70/10/20 split for training/validation/test. Frequencies were computed for the whole cohort.

#### 2.1.3. Handling Class Imbalance

Given the pronounced imbalance in VinBigData (Table 3), we adopt complementary measures to reduce bias toward frequent labels. First, we apply a multi-label stratified splitting strategy to construct the 70/10/20 partitions, which helps preserve both marginal label prevalence and typical co-occurrence patterns across train/validation/test subsets.

**Figure 3:**
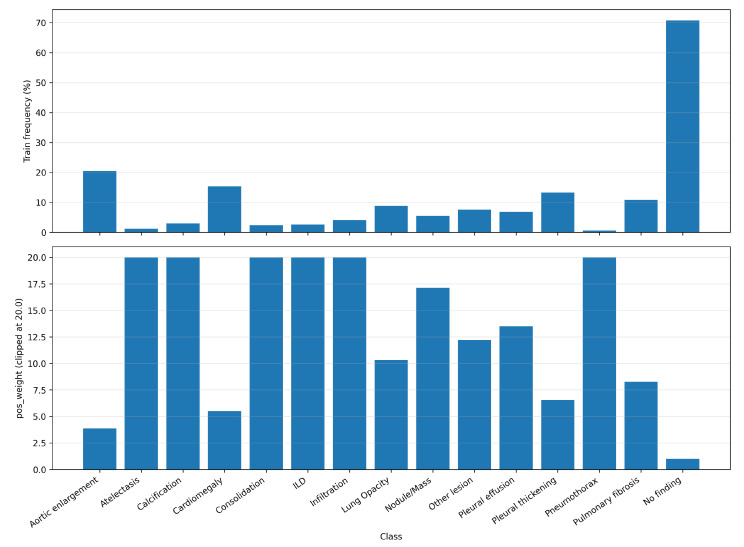
Training-set label frequency (**top**) and the corresponding positive-class weights (pos_weight) used in the weighted BCE loss (**bottom**). Rare labels receive larger weights to counter class imbalance; weights are clipped to a maximum value (20) to avoid unstable gradients.

Second, we used a class-weighted binary cross-entropy loss by assigning a positive-class weight to each label based on its prevalence in the training split. Specifically, for each class *c* we define the weight $w_{c}$ as in Equation (1):

(1) $$w_{c}=\frac{N_{c}^{-}}{N_{c}^{+}},$$

where $N_{c}^{+}$ and $N_{c}^{-}$ denote the number of positive and negative training samples for class *c*, respectively. These weights are applied in BCEWithLogitsLoss via the pos_weight parameter, increasing the penalty for missed positives in low-prevalence labels. To prevent excessively large gradients for very rare findings, we clip $w_{c}$ to a maximum value (20 in our implementation). Figure 3 shows the relationship between the training-set frequencies and the resulting weights.

Finally, we apply mixup augmentation (Section 2.2) as an additional regulariser, improving robustness in the multi-label setting and mitigating overfitting under class imbalance.

To enable a fair and robust evaluation, we first convert the bounding-box-level annotations into image-level multi-label targets. Specifically, each radiograph is represented by a binary vector $y\in {\left\{0,1\right\}}^{15}$ indicating the presence or absence of each finding. The resulting set of 15,000 labelled images is then partitioned into three disjoint subsets using a multi-label stratified procedure: 70% for training (10,484 images), 10% for validation (1506 images), and 20% for testing (3010 images).

During training, only the training split is used to update the network parameters. The validation split is used exclusively for model selection: it is monitored for early stopping and for selecting the best checkpoint based on validation macro-AUROC. After training is complete, the held-out test split is used once for final reporting. In addition, decision thresholds are tuned on the validation set and then applied unchanged to the test set to prevent information leakage and ensure an unbiased evaluation.

#### 2.1.4. Image Preprocessing and Data Augmentation

The original VinBigData chest X-ray images are stored as DICOM files. Each study is first converted offline to an 8-bit greyscale image by reading the DICOM pixel data, applying the rescale slope and intercept when available, and normalising intensities to the range [0, 255]. For images with MONOCHROME1 photometric interpretation (where higher pixel values correspond to darker display intensities), the greyscale values are inverted so that the lung fields appear bright against a darker background, consistent with standard radiological viewing [28].

**Figure 4:**
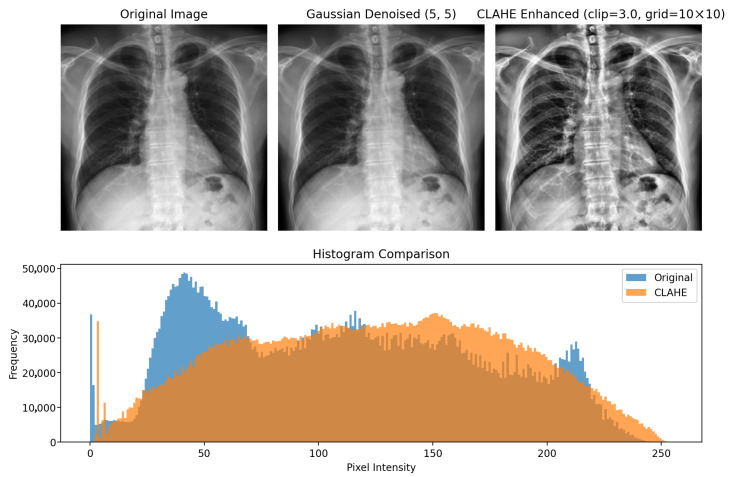
Example VinBigData chest radiograph showing the preprocessing pipeline: original CXR (**left**), Gaussian-denoised image (**middle**), and CLAHE-enhanced image (**right**), with corresponding histogram changes reflecting improved contrast.

To reduce the high-frequency noise and improve the local contrast, we applied a slight Gaussian blur followed by contrast-limited adaptive histogram equalisation (CLAHE). The enhanced images are then resized to 384 × 384 pixels using bilinear interpolation and stored as PNG files, as illustrated in Figure 4.

During training and evaluation, the PNG images were loaded and further processed on the fly. A lightweight CLAHE-based transform was applied as a standardisation step, and images were normalised with the standard ImageNet mean and standard deviation to match the ConvNeXtV2 and ViT-B/16 pretraining configuration. For the training set, we applied mild geometric and photometric augmentations.

- random resized crop to 384 × 384 with scale in [0.85,1.0];
- random horizontal flip;
- small in-plane rotations (±5°);
- light brightness/contrast jitter.

At inference time, we applied a lightweight test-time augmentation (TTA) consisting of a horizontal flip. The final prediction was obtained by averaging the model outputs from the original and flipped views (implemented as logit averaging before the sigmoid), which improved the prediction stability.

### 2.2. Multi-Label Formulation and Loss

The VinBigData chest X-ray abnormalities detection task is inherently multi-label, as a single radiograph may exhibit multiple concurrent findings. The aggregated VinBigData annotations yield, for each radiograph, a binary target vector, as shown in Equation (2).

(2) $$y=\left(y_{1},\ldots ,y_{15}\right)\in {\left\{0,1\right\}}^{15},$$

where $y_{c}=1$ indicates the presence of the *c*-th class (14 abnormalities plus No finding). The hybrid ConvNeXtV2–ViT model outputs, for each input image, a logit vector as in Equation (3).

(3) $$z=\left(z_{1},\ldots ,z_{15}\right)\in R^{15},$$

This vector is transformed into per-class probabilities via the sigmoid function, as shown in Equation (4).

(4) $$p_{c}=\sigma \left(z_{c}\right),\;c=1,\ldots ,15.$$

To account for class imbalance, we employ a weighted binary cross-entropy with logits, where each label is trained with a class-specific positive weight $w_{c}$ estimated from the training split (Section 2.1.3). Therefore, the base classification loss is given by Equation (5).

(5) $$L_{\mathrm{cls}}=\frac{1}{C}\sum_{c=1}^{C}\left[-\;w_{c}\;y_{c}\log\sigma \left(z_{c}\right)-\left(1-y_{c}\right)\log\;\left(1-\sigma (z_{c})\right)\right],\;C=15,$$

which is implemented in practice using BCEWithLogitsLoss(pos_weight).

To incorporate anatomical knowledge, an additional penalty term is defined using the attention map generated by the MXA block in the CNN branch. Let $A\in R^{C_{f}\times H\times W}$ denote the MXA activation tensor, and let $M\in {\left[0,1\right]}^{1\times H\times W}$ be a thoracic mask indicating the lungs and/or heart. Its complement highlights pixels outside the thoracic region. The anatomy-aware penalty is then computed as in Equation (6).

(6) $$L_{\mathrm{anat}}=\lambda \;{∥A⊙\left(1-M\right)∥}_{F},$$

where ⊙ denotes element-wise multiplication, and ${∥\cdot ∥}_{F}$ denotes the Frobenius norm. The final loss is expressed as Equation (7).

(7) $$L=L_{\mathrm{cls}}+L_{\mathrm{anat}}.$$

In our implementation, this formulation is encapsulated in the AnatomyAwareLoss class. For the VinBigData experiments reported here, pixel-level anatomical masks are not available for all images; hence, λ is set to zero, reducing the loss to weighted BCEWithLogitsLoss(pos_weight) while retaining the MXA structure for interpretability [29].

To improve generalisation and mitigate overfitting in the imbalanced multi-label setting, we apply mixup at the batch level. For a mini-batch of images *x* and label vectors *y*, a coefficient as in Equation (8)

(8) λ∼Beta(α,α),

is sampled (with α>0), and a random permutation π of batch indices is generated. The mixed batch is then constructed as in Equation (9).

(9) $$x^{\prime }=\lambda x+\left(1-\lambda \right)x_{\pi },\;y^{\prime }=\lambda y+\left(1-\lambda \right)y_{\pi }.$$

Because the loss is computed with BCEWithLogitsLoss, it naturally supports these soft targets $y^{\prime }$. In practice, we set α=0.2 and apply mixup with probability p=0.3.

### 2.3. Enhanced Hybrid Architecture for Feature Extraction and Classification

The proposed model integrates a deep convolutional backbone (ConvNeXtV2-Base) and a Vision Transformer branch (ViT-B/16) within a unified, end-to-end hybrid design. The motivation is to combine the fine-grained spatial sensitivity of convolutional networks with the global contextual reasoning of transformer encoders, yielding an architecture that captures both local radiographic details and high-level semantic dependencies across the chest field.

The convolutional path follows the ConvNeXtV2-Base architecture, which processes the input CXR $x\in R^{3\times 384\times 384}$ through a sequence of convolutional stages to produce a feature map $F_{\mathrm{cnn}}\in R^{C_{f}\times H\times W}$. A lightweight MXA block, composed of a channel-attention branch and a spatial-attention branch, refines these features into $A_{\mathrm{cnn}}\in R^{C_{f}\times H\times W}$, emphasising radiologically important regions (e.g. lung fields, cardiac silhouette, and pleural line). A global average pooling (GAP) layer then compresses $A_{\mathrm{cnn}}$ into a compact CNN representation $v_{\mathrm{cnn}}\in R^{C_{f}}$.

In parallel, a ViT-Base/16 transformer processes the same input image. The image is decomposed into non-overlapping 16×16 patches, each projected into a *D*-dimensional embedding. A learnable class token $x_{\mathrm{cls}}$ is prepended and positional encodings are added, forming the input sequence shown in Equation (10).

(10) $$Z_{0}=\left[x_{\mathrm{cls}};E_{p_{1}};E_{p_{2}};\ldots ;E_{p_{N}}\right]\in R^{(N+1)\times D}.$$

This sequence is propagated through *L* transformer encoder layers consisting of multihead self-attention and feed-forward blocks with residual connections. The final class token encodes a global semantic summary of the radiograph, denoted $v_{\mathrm{vit}}\in R^{D}$.

The modality-specific vectors $v_{\mathrm{cnn}}$ and $v_{\mathrm{vit}}$ are concatenated to form the hybrid embedding defined in Equation (11), which unifies local texture cues and global contextual information.

(11) $$v_{\mathrm{hyb}}=\left[\;v_{\mathrm{cnn}}\;∥\;v_{\mathrm{vit}}\;\right]\in R^{C_{f}+D}.$$

A linear classification head then maps this hybrid representation to the 15 VinBigData logits, as given in Equation (12),

(12) $$z=Wv_{\mathrm{hyb}}+b,\;z\in R^{15},$$

followed by a sigmoid activation to obtain per-class probabilities. A concise summary of the main components and their roles is provided in Table 4.

### 2.4. Training and Optimisation Details

The hybrid ConvNeXtV2–ViT model is implemented in PyTorch and trained on a single NVIDIA GPU. All experiments use a fixed configuration and controlled random seeds to support reproducibility.

We optimise the model parameters using the AdamW optimiser with a base learning rate of $10^{-4}$ and a weight decay of $10^{-5}$. Training is performed for up to 50 epochs with automatic mixed precision (AMP) enabled to improve computational efficiency.

To stabilise the early training phase and encourage smooth convergence, we adopt a cosine learning-rate schedule with warm-up. The learning rate is linearly increased during the first three epochs and then annealed according to a cosine schedule over the remaining epochs. A mini-batch size of 16 is used together with gradient accumulation (4 steps) to achieve an effective batch size of 64 images per update.

Regarding initialisation and fine-tuning, both the ConvNeXtV2-Base backbone and the ViT-Base/16 branch are initialised from ImageNet-pretrained weights using the timm library. The hybrid network is fine-tuned end-to-end on VinBigData (no layers are frozen). When supported by the backbone implementation, gradient checkpointing is enabled to reduce memory overhead.

In addition, we maintain an exponential moving average (EMA) of the model parameters during training, updated after each optimisation step with a decay factor of 0.9995. At evaluation time, we use the EMA weights rather than the raw weights, which yields more stable predictions and slightly improved validation macro-AUROC.

Table 5 summarises the full training configuration and hyperparameters used in all experiments.

#### Uncertainty Estimation for AUROC

To quantify the uncertainty of AUROC estimates due to the finite size of the held-out test set, we report non-parametric bootstrap confidence intervals. Specifically, we resample test images with replacement (image-level bootstrap) and recompute per-class AUROC, macro-AUROC (mean of per-class AUROCs), and micro-AUROC (flattened labels and probabilities) for each bootstrap replicate. We use 2000 bootstrap replicates and report the bootstrap standard error and percentile-based 95% confidence intervals (2.5th–97.5th percentiles). This procedure estimates evaluation-set variability for a fixed trained model and complements the point estimates reported in the main results tables.

### 2.5. Segmentation-Aware Grad-CAM for Anatomical Localisation

A segmentation-aware Grad-CAM procedure was used to interpret the hybrid ConvNeXtV2–ViT predictions and to assess whether class-specific activations fell within clinically plausible thoracic regions. The procedure produces heatmaps for a target class, combines them with post hoc anatomical masks, and enables both quantitative localisation analysis and ontology-based reasoning (Section 2.6).

#### 2.5.1. CAM Generation from Pre- and Post-MXA Features

Class activation maps were computed within the same trained hybrid ConvNeXtV2–ViT model using two alternative feature sources in the ConvNeXtV2 branch to isolate the effect of MXA attention refinement. The pre-MXA setting uses the CNN feature map immediately before MXA, denoted $f_{\mathrm{cnn}}$, while the post-MXA setting uses the attention-refined feature map after MXA, denoted $a_{\mathrm{cnn}}$. All other components (classifier head, inputs, and decision function) remain unchanged.

Let $A\in R^{K\times H\times W}$ denote the chosen activation tensor (either $f_{\mathrm{cnn}}$ or $a_{\mathrm{cnn}}$), and let $y_{c}$ be the logit for class *c*. The Grad-CAM weights are obtained by spatially averaging the gradients, as shown in Equation (13):

(13) $$w_{k}^{c}=\frac{1}{HW}\sum_{i=1}^{H}\sum_{j=1}^{W}\frac{\partial y_{c}}{\partial A_{kij}},$$

and the class-specific activation map is computed as in Equation (14):

(14) $${\mathrm{CAM}}_{c}\left(i,j\right)=\max\;\left(0,\sum_{k=1}^{K}w_{k}^{c}A_{kij}\right).$$

The heatmap is min–max normalised to [0, 1] and upsampled to the input resolution (384 × 384) using bilinear interpolation. In addition to Grad-CAM, Grad-CAM++ is included as a standard baseline and computed from the same feature sources for direct comparison.

#### 2.5.2. Post Hoc Anatomical Masks (CheXmask)

**Figure 5:**
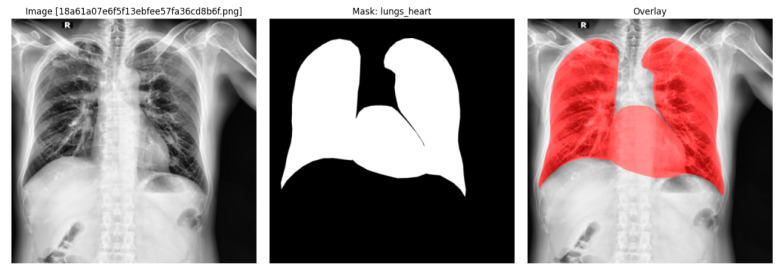
CheXmask anatomical masks used for post hoc localisation analysis. From (**left**) to (**right**): original chest radiograph, lungs and heart binary mask, and mask overlay. The red regions indicate the segmented lung and heart anatomical structures, and the letter “R” denotes the right side of the chest radiograph.

Anatomical reference masks are obtained from CheXmask (VinDr-CXR subset), which provides run-length encoded masks for the left lung, right lung, and heart. These masks are decoded into binary arrays, cached for efficient access, and resized to the network input resolution. A combined mask $M_{\mathrm{lungs}\_\mathrm{heart}}$ is formed by union of lungs and heart masks. These masks are used only for post hoc evaluation and interpretation; no segmentation information is used during training.An example VinBigData radiograph with the corresponding lungs_heart mask and its overlay on the original image is shown in Figure 5.

For region-wise analysis, additional derived masks are computed post hoc: (i) apical and basal lung zones obtained by splitting the lung mask along the vertical axis, and (ii) a pleural band obtained by morphological erosion followed by subtraction to represent the peripheral pleural region.

#### 2.5.3. Top-*p* Localisation and Quantitative Measures

A top-*p* strategy was adopted to obtain a binary localisation region from a continuous heatmap; a top-*p* strategy is adopted. Given a normalised heatmap ${\mathrm{CAM}}_{c}\in {\left[0,1\right]}^{H\times W}$, the top-*p* mask is defined as in Equation (15):

(15) $$M_{\mathrm{top}\text{-}p}\left(i,j\right)=\left\{\begin{array}{cc} 1, & {\mathrm{CAM}}_{c}\left(i,j\right)\geq \tau_{p},\\ 0, & \mathrm{otherwise}, \end{array}\right.$$

where $\tau_{p}$ is the (1−p) quantile of the heatmap values. Unless otherwise stated, p=0.20 is used.

Localisation is quantified using two complementary measures against VinBigData radiologist bounding boxes (when available):

- Mean IoU at top-p: intersection-over-union between $M_{\mathrm{top}\text{-}p}$ (Equation (15)) and the union of class-specific bounding boxes.
- Hit Rate: percentage of cases where $M_{\mathrm{top}\text{-}p}$ (Equation (15)) has any non-zero intersection with the class bounding boxes.

Because Hit Rate counts any overlap as success, it can approach saturation when the top-*p* region is spatially broad; Mean IoU therefore serves as the primary indicator of alignment quality.

To relate activations to anatomical plausibility, an anatomy overlap ratio is also computed as in Equation (16):

(16) $$r_{\mathrm{inside}}=\frac{|M_{\mathrm{top}\text{-}p}\cap M_{\mathrm{lungs}\_\mathrm{heart}}|}{|M_{\mathrm{top}\text{-}p}|},$$

which measures the fraction of the most activated pixels located within the lungs + heart mask.

#### 2.5.4. Evidence Vector for Ontology-Based Reasoning

For each image and target class, the heatmap-derived measurements are summarised into an evidence vector as shown in Equation (17):

(17) $$e=\left(\mathrm{prob},\;\mathrm{inside},\;\mathrm{inside}\_\mathrm{heart},\;\mathrm{apical},\;\mathrm{basal},\;\mathrm{near}\_\mathrm{pleura},\;\mathrm{near}\_\mathrm{heart}\right),$$

where each component is a normalised fraction in [0,1].

The evidence vector in Equation (17) is subsequently converted into fuzzy predicates and combined with ontology-aligned rules to produce an ontology support score and a structured narrative explanation (Section 2.6). Quantitative localisation results are reported in Section 3.2.1, followed by qualitative heatmap examples and anatomy-aware overlays.

### 2.6. Ontology-Based Neuro-Symbolic Reasoning and Fuzzy Rule Layer

On top of the trained hybrid ConvNeXtV2–ViT classifier, a CXR-specific neuro-symbolic layer is implemented, which combines sub-symbolic evidence (multi-label probabilities and Grad-CAM heatmaps) with symbolic knowledge encoded in a clinical decision support system ontology (CXR-CDSS) represented in OWL. For each image, the network outputs a 15-dimensional probability vector $\hat{y}\in {\left[0,1\right]}^{15}$ together with a class-specific Grad-CAM map derived from the MXA block. CheXmask VinDr-CXR segmentations provide lung and heart masks, which are further partitioned into apical and basal lung zones and a thin pleural “shell” band, as described in Section 2.5.2.

For each class *c*, the evidence vector $e_{c}$ is converted into fuzzy predicates using piecewise-linear membership functions ramp_up and ramp_down, such as inside_lungs_high, near_pleura_high, apical_emphasis, basal_emphasis, and prob_very_high. These predicates are combined by a fuzzy rule base that is tailored to each VinBigData class and aligned with the CXR-CDSS ontology. Rules use a product t-norm for conjunction and a probabilistic sum for disjunction. Intuitively, pleural effusion is supported when activation is basal, pleural-based and intrapulmonary with sufficient probability; cardiomegaly is supported when a large fraction of activation overlaps the cardiac silhouette; pneumothorax is supported when activation is apical, pleural and relatively heart-sparing; and parenchymal opacities are supported when activation is intrapulmonary but away from the mediastinum.

For each class *c*, the maximum rule activation is taken as an ontology support score $s_{c}^{\mathrm{ont}}$, quantifying how well the observed spatial pattern matches the radiological pattern encoded in the ontology. The CXR-CDSS ontology organises VinBigData labels and composite concepts (e.g. cardiomegaly_with_effusion) into a hierarchy of thoracic abnormalities. Each dataset label is represented as a subclass of ThoracicAbnormality (or NormalFinding for “No finding”) and associated with typical anatomical locations and imaging patterns, as summarised in Table 6.

### 2.7. Post Hoc Out-of-Distribution Detection

In a deployed setting, a CXR classifier will inevitably receive inputs that differ from the training distribution, for example limb radiographs uploaded by mistake or studies with unusual acquisition characteristics. To mitigate overconfident but unreliable predictions in such cases, a post hoc out-of-distribution (OOD) detection layer is attached to the trained ConvNeXtV2–ViT model. The layer computes four complementary scores: (i) confidence (maximum predicted probability), (ii) mean Bernoulli entropy, (iii) an energy score, and (iv) a Mahalanobis distance in the fused feature space, following standard OOD baselines and energy-/distance-based detectors [23,30,31].

Let $z\in R^{15}$ denote the model logits and $\hat{y}=\sigma \left(z\right)\in {\left[0,1\right]}^{15}$ the per-class probabilities obtained via a sigmoid activation.

#### 2.7.1. Confidence (Max Probability)

Following Hendrycks and Gimpel [30], the confidence score is defined as the maximum predicted probability:

(18) $$s_{\mathrm{conf}}=\max_{c}{\hat{y}}_{c}.$$

Lower values of $s_{\mathrm{conf}}$ in Equation (18) indicate that the model is not confident in any thoracic abnormality label. An OOD flag is raised when $s_{\mathrm{conf}}$ falls below a calibration threshold $\tau_{\mathrm{conf}}$.

#### 2.7.2. Mean Bernoulli Entropy

Because the classifier is multi-label, the Bernoulli entropy is computed for each output and averaged across classes:

(19) $$s_{\mathrm{ent}}=\frac{1}{15}\sum_{c=1}^{15}\left[-{\hat{y}}_{c}\log\left({\hat{y}}_{c}\right)-\left(1-{\hat{y}}_{c}\right)\log\left(1-{\hat{y}}_{c}\right)\right].$$

The entropy score in Equation (19) increases when the model outputs are more uncertain. An OOD flag is raised when $s_{\mathrm{ent}}$ exceeds an upper threshold $\tau_{\mathrm{ent}}$.

#### 2.7.3. Energy Score

We also adopt the energy-based detector of Liu et al. [23]. Using the multi-label logits, the energy score is defined as follows:

(20) $$s_{\mathrm{energy}}=-T\log\sum_{c=1}^{15}\exp\left(z_{c}/T\right),$$

with temperature T=1. In practice, in-distribution CXRs tend to produce more negative energies, whereas OOD inputs yield higher (less negative) values. An image is flagged as OOD if $s_{\mathrm{energy}}$ in Equation (20) is above a threshold $\tau_{\mathrm{energy}}$ estimated from in-distribution calibration data.

#### 2.7.4. Mahalanobis Distance in Fused Feature Space (Diagonal)

To capture how far an input lies from the manifold of training features, a Mahalanobis-type distance is computed in the fused ConvNeXtV2–ViT embedding. A dedicated function extract_fused_features(...) concatenates the MXA-refined CNN representation and the ViT global representation into a vector $f\in R^{D}$. From a calibration subset of in-distribution images, the empirical mean μ and the per-dimension variance vector $\sigma^{2}$ are estimated (diagonal covariance approximation for numerical stability in high-dimensional embeddings). The resulting diagonal Mahalanobis distance is as follows:

(21) $$s_{\mathrm{Maha}}\left(f\right)=\sqrt{\sum_{i=1}^{D}\frac{{\left(f_{i}-\mu_{i}\right)}^{2}}{\sigma_{i}^{2}+\epsilon }},$$

where ϵ is a small regularizer. An OOD flag is raised when $s_{\mathrm{Maha}}$ in Equation (21) exceeds a threshold $\tau_{\mathrm{Maha}}$.

#### 2.7.5. Calibration and Combined Decision

All thresholds are computed *post hoc* on in-distribution validation images (5th percentile for $s_{\mathrm{conf}}$, and 95th percentile for $s_{\mathrm{ent}}$, $s_{\mathrm{energy}}$, and $s_{\mathrm{Maha}}$). The final combined detector uses an OR rule:

(22) $$\mathrm{OOD}\Leftrightarrow \left(s_{\mathrm{conf}}<\tau_{\mathrm{conf}}\right)\;\vee \;\left(s_{\mathrm{ent}}>\tau_{\mathrm{ent}}\right)\;\vee \;\left(s_{\mathrm{energy}}>\tau_{\mathrm{energy}}\right)\;\vee \;\left(s_{\mathrm{Maha}}>\tau_{\mathrm{Maha}}\right).$$

The decision rule in Equation (22) operates on the multi-label outputs and the shared fused embedding without assuming mutual exclusivity among classes. This OOD layer operates on a fixed trained checkpoint and does not require retraining.

#### 2.7.6. OOD Benchmark (Non-Thoracic Radiographs)

To evaluate OOD detection under realistic “wrong-study” scenarios, a non-chest OOD set is created by randomly sampling 400 limb radiographs from the public Kaggle dataset *Bone Fracture Multi-Region X-ray Data* (fractured and non-fractured). This benchmark is used only for evaluation and is never used for training or threshold calibration.

#### 2.7.7. Reproducible OOD Evaluation Protocol

OOD thresholds are calibrated on 400 in-distribution images sampled from the fixed validation split (vinbig_val_10.csv) using a fixed random seed. We then evaluate OOD detection on a balanced benchmark consisting of 400 ID validation images and 400 non-thoracic bone radiographs, where the OOD file list is generated with the same seed and saved for traceability. Table 7 summarises the complete OOD evaluation configuration, including detector definitions, quantile-based threshold rules, and the OR fusion decision used in this work.

## 3. Results

### 3.1. Overall Multi-Label Detection Performance on VinBigData

This section reports the main classification results on the VinBigData chest radiograph cohort. As described in Section 2.1, the official annotation file is provided at bounding-box level and is first converted to image-level multi-label targets. The final dataset contains 15,000 CXRs and is split at the image level using the predefined 70/10/20 protocol: 70% for training, 10% for validation (model selection, early stopping, and threshold fitting), and 20% as a held-out test set for final reporting. The model is trained only with image-level labels; bounding boxes are used solely for qualitative comparison with Grad-CAM and are not used as direct supervisory signals.

ConvNeXtV2-Base was selected as the CNN branch for local feature extraction based on empirical benchmarking and architecture suitability. Using the same split and evaluation protocol, we evaluated a diverse set of CNN baselines (VGG16, ResNet50, DenseNet121, InceptionV3, EfficientNet-B4, and ConvNeXtV). Among these CNN-only models, ConvNeXtV achieved the strongest test macro-AUROC (Table 8), motivating the use of a ConvNeXt-style local branch in the proposed hybrid. Architecturally, ConvNeXtV2 preserves convolutional inductive bias for localised radiographic patterns while adopting optimisation characteristics that align well with transformer-style training, making it a stable local branch for fusion with ViT representations.

**Figure 6:**
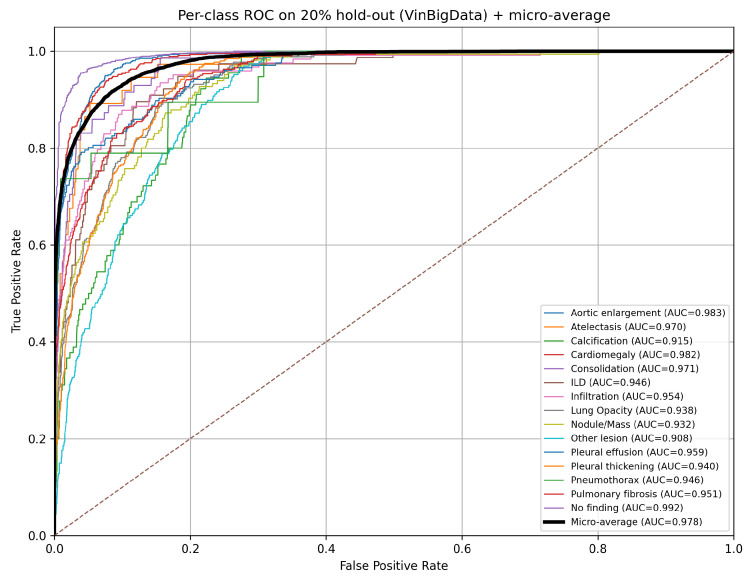
Per-class ROC curves on the 20% hold-out test split of VinBigData. The bold black curve indicates the micro-averaged ROC (micro-AUROC = 0.9777). The dashed diagonal line represents chance-level performance (AUROC = 0.5).

Early stopping and model selection are performed using the macro-averaged AUROC (macro-AUROC) on the validation split. We emphasise AUROC because it is threshold-independent and is a standard discrimination metric for multi-label CXR benchmarks under class imbalance. On the held-out 20% test split, the best checkpoint achieves Macro-AUROC = 0.9525 and Micro-AUROC = 0.9777 (Figure 6). For completeness at a fixed operating point, we additionally report threshold-dependent decision metrics (precision and F1-score) computed using per-class thresholds tuned on the validation split and applied unchanged to the test split to prevent data leakage. The detailed per-class results are presented in Table 9. Overall, the decision-level performance remains high (macro-F1 = 93.66%, micro-F1 = 95.92%). Lower F1-scores for heterogeneous and/or lower-prevalence findings (e.g., Other lesion) are expected because F1 is threshold-dependent and sensitive to false positives/negatives, whereas AUROC evaluates ranking performance across all thresholds.

To quantify uncertainty due to the finite size of the held-out test set, we additionally report bootstrap standard errors and 95% confidence intervals for macro- and micro-AUROC, reported alongside the main results.

#### 3.1.1. Training Dynamics and Generalisation

**Figure 7:**
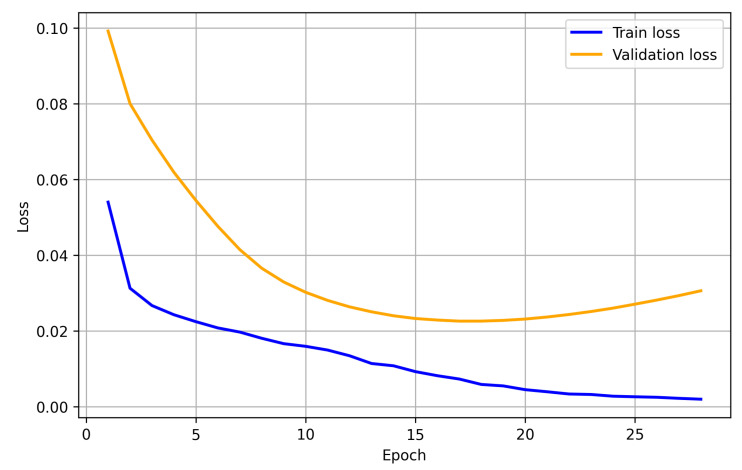
Training (blue) and validation (orange) loss across epochs; early stopping selects the best validation checkpoint (best epoch = 24).

**Figure 8:**
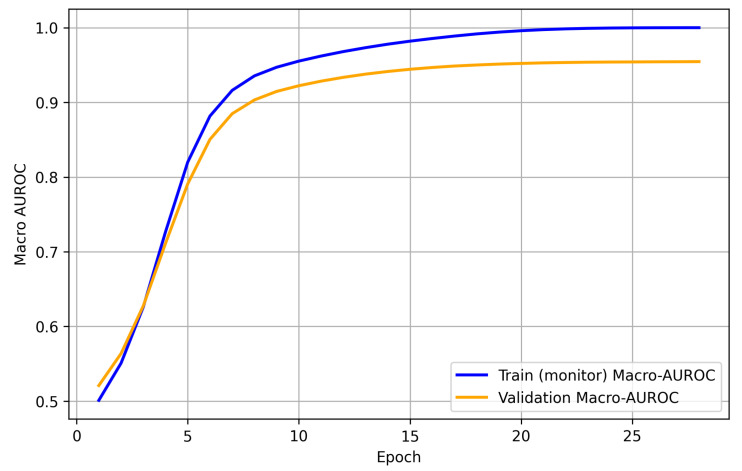
Macro-AUROC across epochs for a fixed training-monitor subset (blue) and the validation split (orange); the best checkpoint is selected at epoch 24.

To assess convergence and generalisation, training was performed with early stopping (patience = 7) and model selection based on the validation macro-AUROC. Training was terminated at epoch 28, with the best checkpoint selected at epoch 24 (best VAL macro-AUROC = 0.9540). As shown in Figure 7 and Figure 8, the training loss decreases monotonically. In contrast, the validation loss dropped rapidly in the early epochs and then plateaued with only a mild late-epoch increase, indicating limited overfitting. In parallel, the validation macro-AUROC rises sharply and then saturates, whereas the training macro-AUROC (computed on a fixed training-monitor subset) remains slightly higher, consistent with stable generalisation.

#### 3.1.2. Comparison to Strong Baseline Architectures and Hybrid Combinations

To provide broader context and align with common evaluation practice in hybrid studies, we compare the proposed model against strong CNN-only baselines from multiple architectural families (VGG16, ResNet50, DenseNet121, InceptionV3, EfficientNet-B4, and ConvNeXtV), a transformer-only baseline (ViT-B/16), and representative CNN–ViT hybrid combinations (ResNet50 + ViT-B/16 and EfficientNet-B4 + ViT-B/16). All models are trained and evaluated under the same predefined 70/10/20 split and identical evaluation protocol.

As shown in Table 8, classical CNN backbones (VGG16 and ResNet50) form a lower baseline cluster, while stronger CNN families (DenseNet121, InceptionV3, EfficientNet-B4) yield consistent improvements. ConvNeXtV provides the best CNN-only performance, and ViT-B/16 achieves the strongest single-branch result overall. Importantly, hybrid fusion improves over either branch alone: both ResNet50 + ViT-B/16 and EfficientNet-B4 + ViT-B/16 outperform their respective CNN-only counterparts, supporting the complementarity of local convolutional cues and global transformer context.

The proposed ConvNeXtV2-Base + ViT-B/16 + MXA achieves the best macro-AUROC on the held-out test split. To further quantify the impact of the CNN branch choice within a hybrid setting, Table 10 reports a per-class AUROC comparison between two representative hybrid baselines (ResNet50 + ViT-B/16 and EfficientNet-B4 + ViT-B/16) and the proposed hybrid.

#### 3.1.3. Test-Time Augmentation (TTA) Visualisation

**Figure 9:**
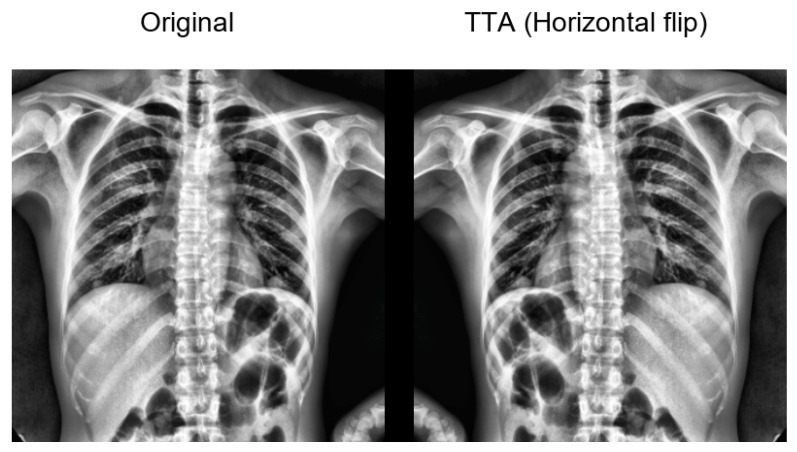
Illustration of the test-time augmentation (TTA) used during evaluation. For each radiograph, we show the original input (**left**) and its horizontally flipped counterpart (**right**). The final prediction is obtained by averaging the model outputs from both views.

To improve transparency and reproducibility of the evaluation protocol, we provide a visual example of the test-time augmentation (TTA) used in our experiments. As illustrated in Figure 9, each test radiograph is evaluated in two inference views: the original image and a horizontally flipped version. The final prediction is obtained by averaging the per-class probabilities from both views, which improves robustness to small spatial variations.

Importantly, the held-out test split is not augmented or expanded: all reported metrics are computed on the original fixed test images. TTA is applied only at inference and therefore does not change the test set or introduce information leakage.

### 3.2. Explainability and Ontology-Based Reasoning

#### 3.2.1. Quantitative CAM Localisation Analysis (VinBigData)

Class activation map (CAM) localisation was assessed on the VinBigData cohort using radiologist bounding-box annotations. Two standard CAM variants were considered: Grad-CAM and Grad-CAM++. To isolate the contribution of the MXA attention refinement, CAMs were computed from two internal feature sources within the same hybrid ConvNeXtV2–ViT model: (i) the pre-MXA CNN feature map before attention refinement ($f_{\mathrm{cnn}}$) and (ii) the post-MXA attention-refined feature map after MXA ($a_{\mathrm{cnn}}$). This design keeps the classifier, input, and decision function unchanged while varying only the feature source used for attribution.

For each class, the CAM was min–max normalised and converted into a binary localisation region by retaining the top p=0.20 (top 20%) activated pixels. The resulting region was compared against the union of class-specific bounding boxes using two localisation metrics: (1) Mean intersection-over-union at top-*p* (Mean IoU) and (2) Hit Rate (%), defined as any non-zero overlap. Results are reported per class, and a macro-average summarises overall localisation behaviour.

Hit Rate is close to saturation across most abnormalities because it counts any overlap as a success, and the top-*p* region at p=0.20 can be spatially broad; even partial contact with a bounding box is sufficient. Mean IoU is therefore the primary indicator of localisation quality, as it reflects the degree of spatial alignment between the CAM region and the annotated abnormality extent.

Across classes, post-MXA explanations show higher Mean IoU than pre-MXA explanations on average (macro Mean IoU: 0.3883 → 0.4311 for Grad-CAM; 0.3710 → 0.4208 for Grad-CAM++), suggesting that MXA refines the spatial concentration of attribution maps towards annotated regions. Larger gains are observed for abnormalities with more consistent or larger spatial extent (e.g., pleural effusion, cardiomegaly), whereas more diffuse or heterogeneous categories (e.g., infiltration, other lesion) remain more challenging for bounding-box alignment, which is reflected in lower IoU values.

The following subsubsection presents qualitative heatmaps and anatomy-aware overlays to visually illustrate the localisation patterns underlying the quantitative scores.

#### 3.2.2. Qualitative Anatomy-Aware Localisation and Neuro-Symbolic Output

Qualitative examples are presented to complement the quantitative localisation results in Table 11. For each selected radiograph, the class-specific Grad-CAM heatmap is visualised and then restricted to the lungs + heart anatomical mask. The inside-mask energy ratio reports the proportion of total CAM activation that lies within the mask, while the remaining activation is counted as outside-mask energy.

#### 3.2.3. Single-Label Case: Aortic Enlargement

**Figure 10:**
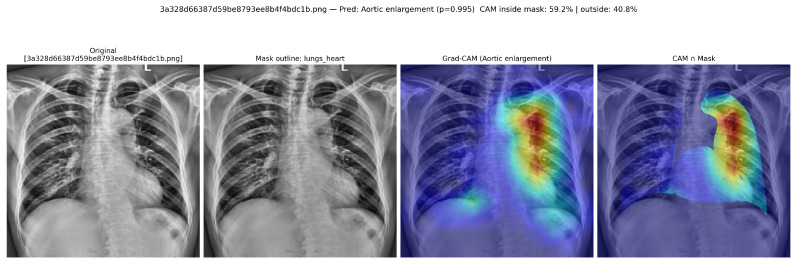
Anatomy-aware Grad-CAM for an aortic enlargement example. From (**left**) to (**right**): original chest radiograph, lungs and heart mask outline, Grad-CAM for the target class, and Grad-CAM restricted to the lungs and heart mask. Color overlays indicate activation intensity. The letter “L” denotes the left side of the chest radiograph. Inside-mask CAM energy is 59.2% (40.8% outside-mask).

**Figure 11:**
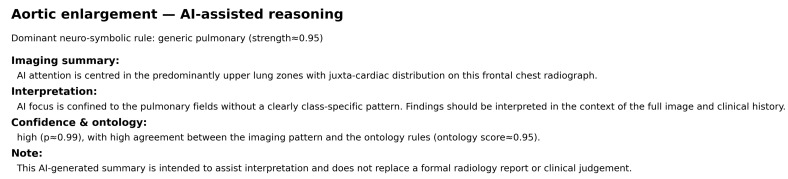
Ontology-aware narrative for the same aortic enlargement example. The reasoning layer summarises the imaging pattern, provides an interpretation, and reports the model probability (p) and the ontology support score.

Figure 10 and Figure 11 illustrate the anatomy-aware Grad-CAM and the corresponding neuro-symbolic narrative for a representative aortic enlargement case. The hybrid ConvNeXtV2–ViT model assigns a high probability to this label (p≈0.995). After intersecting the Grad-CAM map with the CheXmask lungs+heart segmentation, 59.2% of the CAM energy lies inside the anatomical mask and 40.8% lies outside, indicating that the dominant saliency is concentrated over the cardiomediastinal region and adjacent upper lung fields.

**Figure 12:**
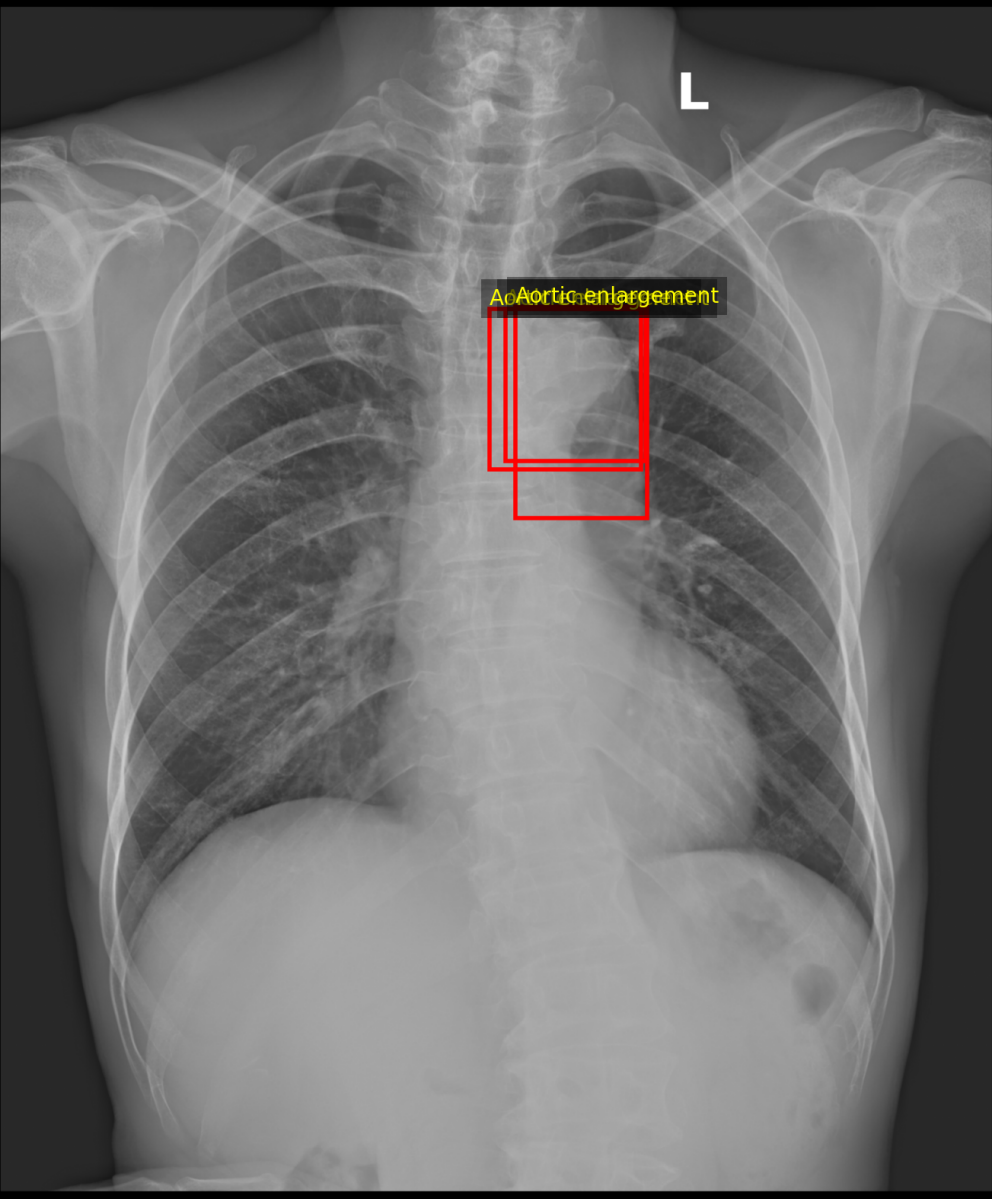
Radiologist bounding-box annotations for the same case, indicating an ectatic aortic contour along the left upper mediastinum. The red frame denotes the expert-annotated region of interest, and the letter “L” indicates the left side of the chest radiograph. The main Grad-CAM hotspot in Figure 10 overlaps this region.

Radiologist bounding boxes (Figure 12) delineate an ectatic aortic contour along the left upper mediastinum. The strongest Grad-CAM activation overlaps this region, supporting spatial concordance between the model attribution and the expert annotation. The neuro-symbolic layer activates a generic pulmonary rule with strength ≈0.95 and generates a structured narrative describing predominantly upper-zone attention with juxta-cardiac distribution and high ontology support (ontology score ≈0.95). Overall, this example illustrates a high-confidence prediction with clinically plausible localisation and a consistent ontology-linked explanation.

#### 3.2.4. Multi-Label Case: Infiltration, Lung Opacity, and Pleural Effusion

**Figure 13:**
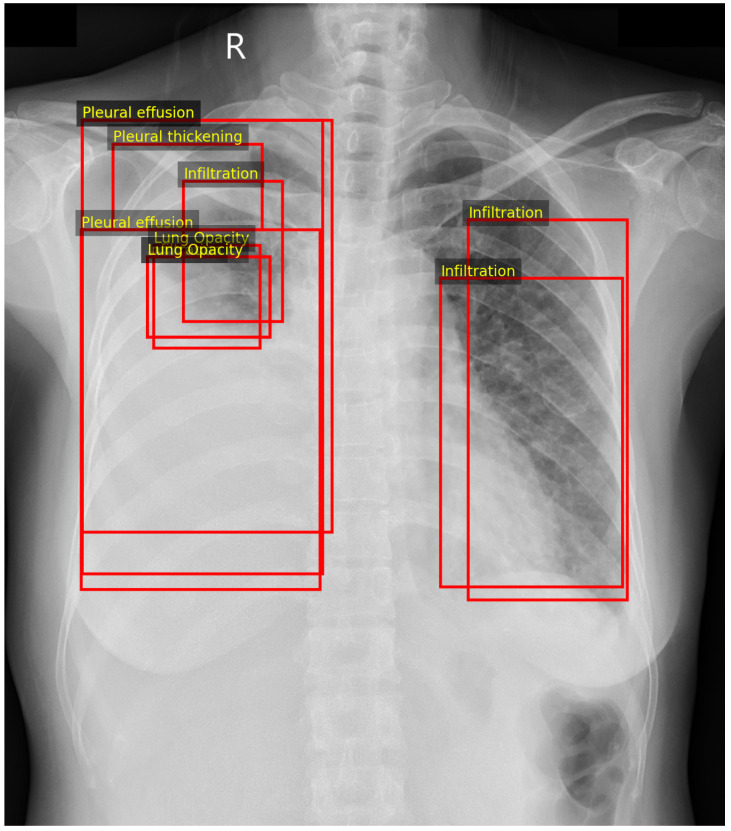
Radiologist bounding-box annotations for the selected multi-label VinBigData study. Red frames denote expert-annotated regions of interest, and the letter “R” indicates the right side of the chest radiograph. Overlapping boxes indicate pleural effusion, pleural thickening, lung opacity, and infiltration, reflecting the coexistence of multiple abnormalities within the same examination.

**Figure 14:**
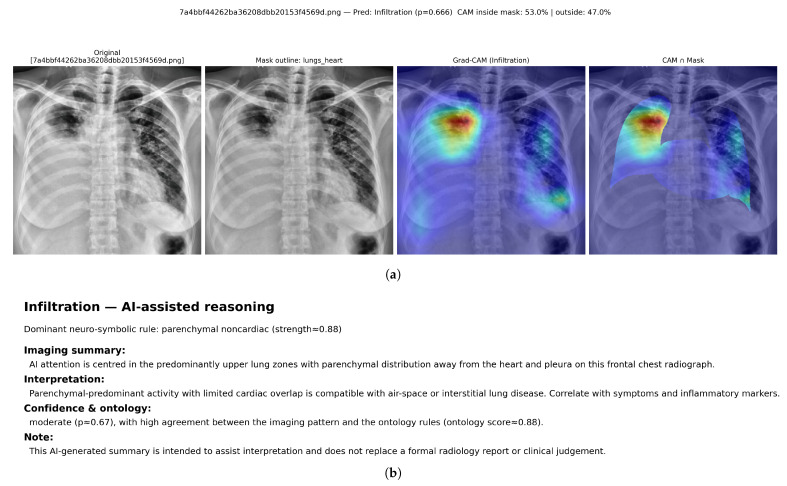
Anatomy-aware Grad-CAM and neuro-symbolic explanation for infiltration in the multi-label case of Figure 13. Color overlays represent Grad-CAM activation intensity, with warmer colors indicating higher relevance to the predicted class. The model probability (p) and ontology support score are reported in the ontology-aware narrative. (**a**) Anatomy-aware Grad-CAM for the infiltration label. (**b**) Ontology-aware narrative for the infiltration label.

**Figure 15:**
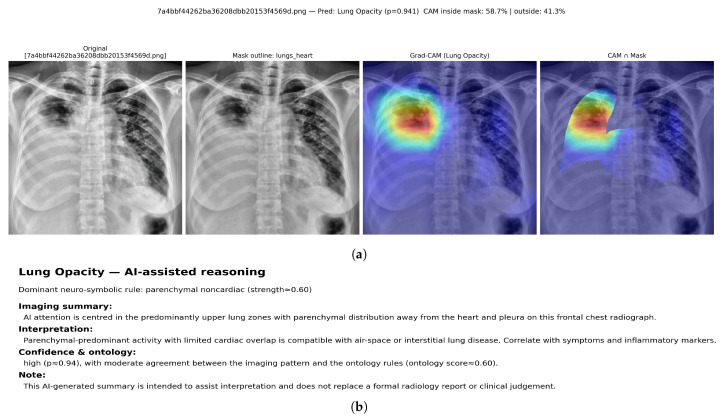
Anatomy-aware Grad-CAM and neuro-symbolic explanation for lung opacity in the multi-label case of Figure 13. Color overlays represent Grad-CAM activation intensity, with warmer colors indicating higher relevance to the predicted class. The model probability (p) and ontology support score are reported in the ontology-aware narrative. (**a**) Anatomy-aware Grad-CAM for the lung opacity label. (**b**) Ontology-aware narrative for the lung opacity label.

**Figure 16:**
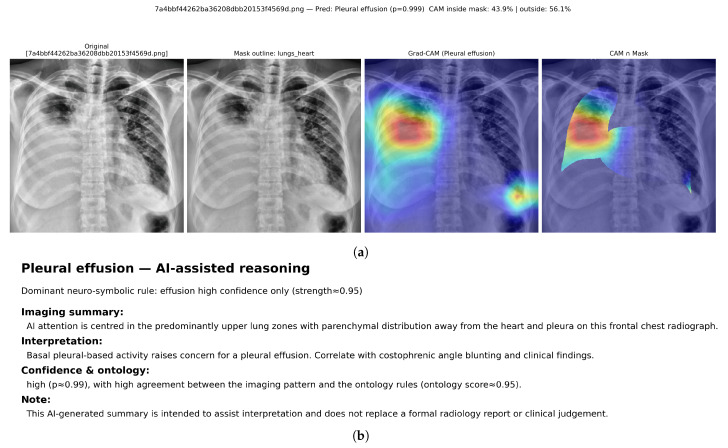
Anatomy-aware Grad-CAM and neuro-symbolic explanation for pleural effusion in the multi-label case of Figure 13. Color overlays represent Grad-CAM activation intensity, with warmer colors indicating higher relevance to the predicted class. The model probability (p) and ontology support score are reported in the ontology-aware narrative. (**a**) Anatomy-aware Grad-CAM for the pleural effusion label. (**b**) Ontology-aware narrative for the pleural effusion label.

Figure 13 shows a multi-label VinBigData study with overlapping radiologist bounding boxes for infiltration, lung opacity, pleural effusion, and pleural thickening. Using a fixed threshold of 0.5, the hybrid model predicts three positive labels: infiltration, lung opacity, and pleural effusion. Label-specific Grad-CAM maps are generated for each predicted label, followed by mask-restricted visualisation and a corresponding neuro-symbolic narrative (Figure 14, Figure 15 and Figure 16).

For infiltration, the Grad-CAM heatmap highlights predominantly right-sided parenchymal regions, with 53.0% of CAM energy inside the lungs+heart mask and 47.0% outside. The reasoning layer selects a parenchymal noncardiac rule with strength ≈0.88 and reports a moderate prediction probability (p≈0.67). For lung opacity, the attribution concentrates over the left upper–mid lung area, with 58.7% inside-mask energy and 41.3% outside-mask energy, while the ontology support is more modest (ontology score ≈0.60) despite a high model probability (p≈0.94). For pleural effusion, the heatmap shifts to the dependent hemithorax with emphasis near the basal pleural region; 43.9% of CAM energy lies inside the mask and 56.1% lies outside, consistent with spill-over toward the diaphragm and sub-diaphragmatic boundary. The reasoning layer activates an effusion-confidence rule with strength ≈0.95 and reports a high probability (p≈0.99).

Together, these outputs demonstrate that a single multi-label radiograph can be decomposed into label-specific spatial patterns with distinct clinical narratives. The heatmaps show overlapping but separable regions aligned with the expert annotations in Figure 13, while the ontology scores differentiate highly confident pleural findings from more ambiguous parenchymal patterns.

#### 3.2.5. Qualitative Example: Obvious OOD Non-Thoracic Radiograph

To illustrate the behaviour of the post hoc OOD layer, we analyse a representative obvious OOD case in which a non-thoracic radiograph (bone/foot X-ray) is intentionally passed through the VinBigData-trained ConvNeXtV2–ViT model. While the classifier outputs No finding, the OOD module correctly rejects the sample as out-of-distribution.

In this example, the maximum predicted probability is low ($s_{\mathrm{conf}}=0.541$), falling below the in-distribution calibration threshold ($\tau_{\mathrm{conf}}=0.768$). The energy score is also elevated relative to typical in-distribution values ($s_{\mathrm{energy}}=-0.937$ vs. $\tau_{\mathrm{energy}}=-1.599$), indicating an atypical logit profile. In addition, the fused-feature distance is large ($s_{\mathrm{Maha}}=71.65$ vs. $\tau_{\mathrm{Maha}}=55.56$), confirming that the representation lies outside the VinBigData chest-X-ray feature manifold. Although the entropy score does not exceed its threshold in this instance ($s_{\mathrm{ent}}=0.2616<\tau_{\mathrm{ent}}=0.3003$), the paper-aligned combined OR rule correctly flags the image as OOD because at least one detector triggers. Overall, this example demonstrates that the proposed post hoc layer can reject anatomically irrelevant inputs and reduce the risk of spurious chest abnormality predictions on images outside the model’s training domain. The quantitative OOD scores and corresponding threshold comparisons for this example are summarised in Table 12.

##### Quantitative OOD Detection Summary (400 ID vs. 400 Non-Chest OOD)

Following calibration on 400 in-distribution validation images, we evaluated OOD detection on a balanced benchmark of 400 VinBigData images (ID) and 400 non-thoracic bone radiographs (OOD). To provide a single, easy-to-interpret summary as requested by the reviewer, Table 13 reports AUROC, AUPRC, and FPR95 for each detector, together with the operating thresholds (τ) used by the proposed OR-based decision rule. Here, FPR95 denotes the ID false-positive rate at the threshold where the OOD true-positive rate (TPR) equals 0.95. Across all detectors, separability is strong, with Mahalanobis distance providing the best overall discrimination (Table 13). Confidence, entropy, and energy also achieve consistently high AUROC/AUPRC values, with corresponding FPR95 values reported in Table 13. Under the paper-aligned combined OR decision at the calibrated operating thresholds, the system achieves perfect OOD recall with a low ID false-positive rate; the corresponding confusion matrix and derived operating-point metrics (accuracy, precision, recall, and F1) are summarised in Table 14.

### 3.3. Generalisation to NIH ChestX-ray14 and CheXpert

To evaluate cross-dataset generalisation, we further assessed the proposed ConvNeXtV2–ViT model on the NIH ChestX-ray14 (NIH14) dataset [32], which contains 112,120 frontal chest radiographs annotated with 14 thoracic findings plus a No Finding label. Following the patient-wise protocol described in Section 2, the model was trained on the NIH14 training split (78,629 images), thresholds were tuned on the validation split (11,050 images), and final performance was reported on a held-out test split (22,441 images).

Table 15 reports per-class AUROC values on the NIH14 validation and test sets. The model achieves a macro-AUROC of 0.9102 on validation and 0.9106 on test, indicating strong generalisation to this larger and noisier cohort. Performance is highest for well-defined abnormalities such as *Emphysema*, *Hernia*, *Cardiomegaly*, *Pneumothorax*, *Effusion*, and *Mass*, whereas diffuse or visually ambiguous entities such as *Infiltration*, *Consolidation*, and *Pneumonia* remain more challenging, consistent with prior NIH14 reports. Overall, the macro-AUROC decreases by less than 4% relative to VinBigData (≈0.9525), supporting robust cross-cohort transferability.

**Figure 17:**
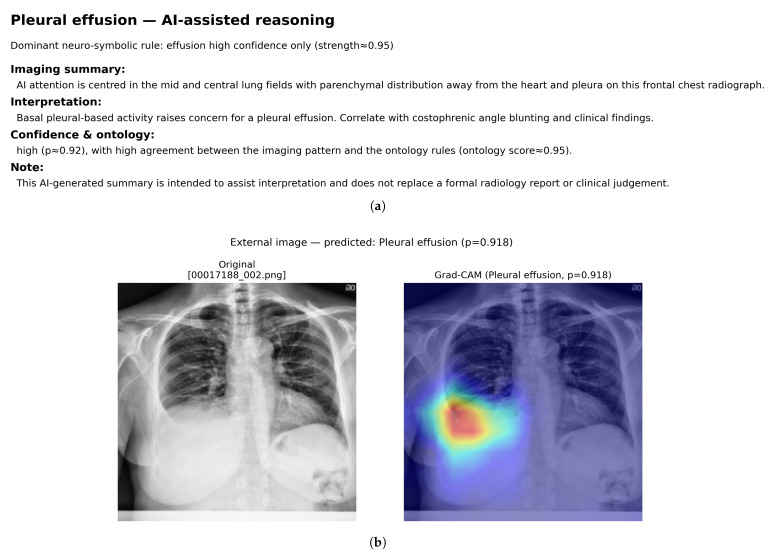
External NIH ChestX-ray14 example predicted as pleural effusion. Color overlays represent Grad-CAM activation intensity, with warmer colors indicating higher relevance to the predicted class. The model probability (p) and ontology support score are reported in the ontology-aware narrative. (**a**) AI-generated ontology-aware reasoning describing basal pleural-based activity and correlating imaging findings with ontology scores. (**b**) Original radiograph with Grad-CAM overlay (p = 0.918), showing activation concentrated over the dependent basal hemithorax, consistent with an effusion pattern.

In addition to quantitative evaluation, qualitative analysis confirmed that Grad-CAM and ontology reasoning remain informative on NIH14 images. For a representative external case of Pleural effusion, the model predicted a high probability (p = 0.918); the Grad-CAM map highlighted the dependent basal hemithorax, and the neuro-symbolic layer produced an effusion-focused narrative with high ontology support. The two panels are presented in Figure 17—panel (a) shows the reasoning text, and panel (b) the radiograph with its Grad-CAM overlay.

We also assessed generalisation on the CheXpert validation set, restricted to frontal radiographs and labels overlapping with the VinBigData task. On this subset, the model achieves a macro-AUROC of 0.849, with strong results for *Consolidation* (0.928), *Effusion* (0.907), *Edema* (0.874), and *Atelectasis* (0.872). *Pneumonia* and *Pneumothorax* remain more difficult (0.806 and 0.757, respectively), consistent with the known label noise and imbalance in CheXpert. Several labels (e.g., *Emphysema*, *Fibrosis*, *Hernia*, *Mass*, *Nodule*, and *Pleural thickening*) were degenerate (all positives or all negatives) and thus excluded from AUROC computation. The retained classes are summarised in Table 16.

## 4. Discussion

This study presented a hybrid ConvNeXtV2–ViT model for multi-label chest X-ray (CXR) analysis on VinBigData and integrated three practical system-level modules: (i) segmentation-aware Grad-CAM for anatomical localisation, (ii) ontology-based reasoning for producing structured explanations and support scores, and (iii) post hoc out-of-distribution (OOD) detection as an additional safety layer. Below we discuss the major findings, generalisation behaviour, and clinical implications.

On the predefined 70/10/20 split of VinBigData (15,000 images), the proposed hybrid model achieves strong performance on the held-out test set with macro-AUROC = 0.9525 and micro-AUROC = 0.9777 (Table 9, Figure 6). Most classes obtain high AUROC values, including clinically important findings such as cardiomegaly, aortic enlargement, pleural effusion, consolidation, and infiltration. These results suggest that combining a modern CNN backbone (ConvNeXtV2) with a ViT branch, together with standard regularisation (mixup), EMA evaluation, and a warm-up+cosine schedule, provides robust multi-label performance without requiring bounding boxes during training.

When evaluated on external datasets, performance decreases as expected due to differences in patient populations, acquisition conditions, and labelling schemes. Nevertheless, the model remains in a competitive range on NIH ChestX-ray14 (macro-AUROC ≈ 0.91 on the test split) and on the CheXpert validation set (macro-AUROC ≈ 0.85). The gap relative to VinBigData highlights the importance of domain shift in CXR modelling and motivates further work on systematic domain adaptation and calibration.

The segmentation-aware Grad-CAM analysis, supported by CheXmask anatomical masks, provides clinically plausible localisation patterns in both single-label and multi-label cases (Figure 10, Figure 11, Figure 12, Figure 13, Figure 14, Figure 15 and Figure 16). Instead of producing only heatmaps, the system quantifies how much activation falls inside lungs/heart and how it is distributed across apical, basal, and pleural regions. These localisation measurements are then converted into fuzzy predicates and combined with model probabilities through ontology-aligned rules to generate class-specific support scores and short textual summaries. Importantly, support scores can differ from raw probabilities, which may help users judge whether a prediction is anatomically convincing (e.g., a high probability with weak/implausible localisation yields reduced support).

To address safety-related concerns, we attached a post hoc OOD module to the trained checkpoint and evaluated it quantitatively using matched-size in-distribution (ID) versus OOD cohorts. Specifically, we scored 400 VinBigData validation images (ID) and 400 non-chest bone/limb radiographs sampled from the public Kaggle dataset *Bone Fracture Multi-Region X-ray Data* (OOD). Across the four post hoc detectors, separation was strong (confidence AUROC = 0.9636, entropy AUROC = 0.9646, energy AUROC = 0.9663, and Mahalanobis AUROC = 0.9910; corresponding AUPRC values were also high). Using percentile-based thresholds calibrated on the ID validation data and a combined OR rule (flagging OOD if any detector triggers), the operating point yielded TN = 386, FP = 14, FN = 0, and TP = 400, corresponding to ID-FPR = 0.035 and OOD-TPR = 1.00, with precision = 0.9662 and F1 = 0.9828. These results support the utility of a lightweight OOD layer for rejecting clear distribution mismatches (e.g., non-thoracic radiographs uploaded by mistake) without retraining the classifier.

Although an anatomy-aware penalty term was defined to discourage attention outside thoracic regions, anatomical masks are not available for all VinBigData images. Therefore, for all reported VinBigData experiments we set λ=0, reducing the training objective to weighted BCEWithLogitsLoss(pos_weight). Nevertheless, anatomy-aware optimisation remains a promising direction for future work when reliable lung/heart masks are consistently available (e.g., in cohorts with provided segmentations). Enabling this term may improve localisation consistency and help reduce anatomically implausible activations.

Future work will include prospective evaluation with expert readers, broader domain-shift experiments, and extending OOD testing to additional cohorts and OOD types. In addition, when consistent masks are available, anatomy-aware optimisation can be enabled during training. The ontology layer can be expanded and partially learned (e.g., tuning membership functions and rule weights based on observed localisation patterns), and incorporating temporal priors and basic clinical metadata may further support richer neuro-symbolic clinical decision support.

## 5. Conclusions

We proposed a hybrid ConvNeXtV2–ViT model for multi-label abnormality detection on chest X-rays and integrated three practical extensions: segmentation-aware Grad-CAM with anatomical masks, ontology-based reasoning for structured explanations and support scores, and post hoc OOD detection as a safety layer. On the predefined 70/10/20 VinBigData split, the model achieves strong discrimination on the held-out test set (macro-AUROC = 0.9525; micro-AUROC = 0.9777). External evaluation on NIH ChestX-ray14 and CheXpert shows the expected performance drop under dataset shift but remains within a useful range, supporting the robustness of the learned representation.

Beyond reporting classification metrics alone, the system provides label-specific localisation maps and anatomy-informed reasoning outputs that connect predicted findings to clinically meaningful regions (lungs, heart, apical/basal zones, and pleural areas). In addition, a quantitative OOD benchmark using matched-size ID vs. OOD cohorts demonstrates strong separation and low ID false-positive rate at a fixed operating point (e.g., ID-FPR = 0.035 with OOD-TPR = 1.00 under the combined rule), indicating that lightweight post hoc OOD scoring can improve safety by flagging clearly mismatched inputs.

This study has limitations: all evaluations are retrospective, CheXmask masks are model-generated, and ontology rules were hand-crafted for the studied labels. The anatomy-aware loss is defined but was not used in the reported VinBigData validation experiments (λ=0 due to incomplete mask availability). Future work will include (i) exploring anatomy-aware training objectives when reliable masks are available, (ii) expanding the ontology and learning parts of the reasoning layer, and (iii) broader OOD testing and prospective clinical evaluation with radiologist feedback. Overall, the results suggest that hybrid CNN–transformer architectures combined with anatomy-aware explainability, neuro-symbolic reasoning, and quantitative OOD safeguards can provide a more transparent and clinically relevant alternative to pure black-box CXR classifiers.

## Figures and Tables

**Table 1:** Summary of selected related studies on chest X-ray analysis and decision support.

Author	Dataset(s)	Methodology	Data Preprocessing	Pros	Cons	XAI/OOD/Ontology
Ait Nasser et al. [18]	Multiple public CXR datasets	Review of CNN-based models for multi-label CXR classification	Not applicable (review)	Comprehensive overview of models, metrics, and challenges	No single model; limited OOD or ontology discussion	XAI: discussed; OOD: limited; Ontology: no
Ashraf et al. [8]	ChestX-ray14 (multi-label)	Ensemble of CNN, ViT, and hybrid backbones (e.g., CoAtNet)	Standard resizing, normalisation, augmentation	Strong AUROC; hybrid ensembles capture local+global context	High computational cost; complex training; no ontology/OOD	XAI: limited; OOD: no; Ontology: no
Yulvina et al. [19]	TB anomalies (NIH subset)	Hybrid CNN–ViT with focal loss and class weighting	Resizing, augmentation, class balancing	Improves TB detection; handles imbalance	Small dataset; qualitative XAI; no OOD/ontology	XAI: Grad-CAM; OOD: no; Ontology: no
Fu et al. [20]	COVID-19 CXR, ChestX-ray14	LungMaxViT hybrid transformer with CNN blocks and MaxViT modules	CLAHE, denoising, augmentation	High AUC and visual explainability	Focus on multiclass; no OOD/ontology reasoning	XAI: Grad-CAM; OOD: no; Ontology: no
Nawaz et al. [21]	VinBigData CXR abnormalities	YOLOv5-based detection for 14 thoracic abnormalities	Resizing, normalistion, standard Kaggle split	Strong lesion localisation and classification	Detection-only; lacks global multi-label view	XAI: no; OOD: no; Ontology: no
Selvaraju et al. [11]	Vision benchmarks	Grad-CAM: class activation mapping for CNNs	Not applicable	Model-agnostic interpretability	Coarse heatmaps; no clinical context	XAI: yes; OOD: no; Ontology: no
Kansal et al. [22]	Public CXR datasets	Transformer-based classifier with Grad-CAM explanation	Standard preprocessing, augmentation	Better accuracy vs. CNNs; interpretable Grad-CAM	Qualitative only; lacks anatomy/OOD integration	XAI: Grad-CAM; OOD: no; Ontology: no
Liu et al. [23]	Generic image classification	Energy-based OOD detection using logits	Standard preprocessing; train/val/test split	Principled OOD scoring; robust thresholding	Not tailored to medical domain; no ontology	XAI: no; OOD: yes; Ontology: no
Budovec et al. [24]	Radiology Gamuts Ontology	Ontology-based decision support for radiology	Ontology mapping of findings and diagnoses	Encodes causal relations between findings and diseases	Structured data only; no deep learning integration	XAI: concept-level; OOD: no; Ontology: yes
Jing et al. [25]	Clinical decision support systems	Review of ontologies in CDSS (SNOMED CT, RadLex, etc.)	Not applicable (review)	Highlights ontology use for medical reasoning	No imaging integration or XAI connection	XAI: indirect; OOD: no; Ontology: yes

**Table 2:** Example multi-label target vectors for the three VinBigData radiographs shown in Figure 2.

Example (CXR)	Positive Labels ($y_{c}=1$)
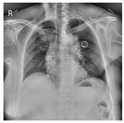	(a) Aortic enlargement; Cardiomegaly; Pulmonary fibrosis
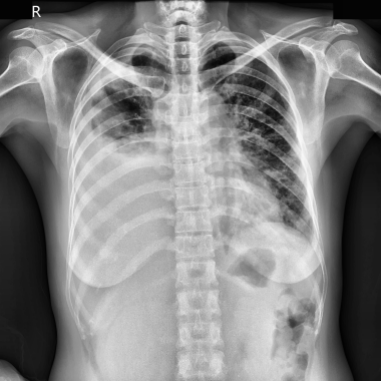	(b) Aortic enlargement; Infiltration; Lung opacity; Pleural effusion; Pleural thickening
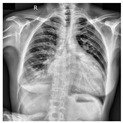	(c) Cardiomegaly; Lung opacity

**Table 3:** Summary of class distribution (number of positive images per label) in the VinBigData CXR dataset.

Class	Train (70%)	Val (10%)	Test (20%)	Total	Frequency (%)
Aortic enlargement	2147	307	613	3067	20.45
Atelectasis	130	19	37	186	1.24
Calcification	317	45	90	452	3.01
Cardiomegaly	1610	230	460	2300	15.33
Consolidation	247	35	71	353	2.35
ILD	270	39	77	386	2.57
Infiltration	429	61	123	613	4.09
Lung Opacity	926	132	264	1322	8.81
Nodule/Mass	578	83	165	826	5.51
Other lesion	794	113	227	1134	7.56
Pleural effusion	723	103	206	1032	6.88
Pleural thickening	1387	198	396	1981	13.21
Pneumothorax	67	10	19	96	0.64
Pulmonary fibrosis	1132	162	323	1617	10.78
No finding	7424	1061	2121	10,606	70.71

**Table 4:** Summary of the enhanced hybrid ConvNeXtV2–ViT architecture for VinBigData CXR classification.

Component	Description/Role
ConvNeXtV2-Base backbone	Hierarchical CNN extracting low- to high-level visual features, producing $F_{\mathrm{cnn}}\in R^{C_{f}\times H\times W}$.
MXA block	Dual attention (channel + spatial) refining $F_{\mathrm{cnn}}$ into $A_{\mathrm{cnn}}$, enhancing pathology-relevant channels and spatial locations.
GAP layer	Global average pooling compresses $A_{\mathrm{cnn}}$ into $v_{\mathrm{cnn}}\in R^{C_{f}}$.
ViT-B/16 branch	Transformer encoder operating on 16×16 patches; the final class token $v_{\mathrm{vit}}$ captures global semantic context.
Fusion layer	Concatenation $v_{\mathrm{hyb}}=\left[v_{\mathrm{cnn}}\;∥\;v_{\mathrm{vit}}\right]$ merges local and global features.
Classifier head	Linear projection from $v_{\mathrm{hyb}}$ to logits $z\in R^{15}$, followed by sigmoid.

**Table 5:** Training configuration and hyperparameters for the ConvNeXtV2–ViT + MXA multi-label model.

Category	Setting
Dataset	VinBigData (train.csv aggregated to image-level multi-label targets)
Split protocol	Predefined 70/10/20 train/validation/test split (fixed CSVs)
Random seed	42 (Python 3.10, NumPy 1.26, PyTorch 2.1, CUDA 11.8)
Input resolution	384 × 384
Backbones	ConvNeXtV2-Base (convnextv2_base) + ViT-B/16-384
Pretraining	ImageNet-pretrained weights (timm), fine-tuned end-to-end
Drop-path	0.10
Fusion	Concatenation of CNN GAP feature + ViT global token feature
Classifier	Linear layer (fused-dim → 15 logits)
Attention module	MXA: Channel attention (ratio = 16) + Spatial attention (kernel = 7)
Loss	BCEWithLogitsLoss (multi-label)
Optimiser	AdamW
Base learning rate	$1\times 10^{-4}$
Weight decay	$1\times 10^{-5}$
Epochs (max)	50
Batch size	16
Gradient accumulation	4 (effective batch size = 64)
Mixed precision	AMP enabled
LR schedule	Linear warm-up (3 epochs) + cosine decay
Early stopping	Patience = 7, min-delta = 0.001
EMA	Enabled, decay = 0.9995 (EMA weights used for evaluation)
Mixup	Enabled, α=0.2, applied with probability 0.3
Test-time augmentation	Horizontal flip (logit averaging before sigmoid)
Workers	0

**Table 6:** Mapping between VinBigData labels and ontology concepts in the proposed CXR-CDSS ontology. Each dataset label is represented as a subclass of ThoracicAbnormality (or NormalFinding for “No finding”) and associated with typical anatomical locations and imaging patterns.

Dataset Label	OWL Class Name	Superclass	Typical Location/Imaging Pattern
Aortic enlargement	AorticEnlargement	ThoracicAbnormality	Located at *Aorta*; associated with *AorticContourEnlargementPattern*.
Atelectasis	Atelectasis	ThoracicAbnormality	Located in *Lung*; associated with *VolumeLossPattern*.
Calcification	Calcification	ThoracicAbnormality	May represent *CalcifiedGranuloma* or other *CalcifiedLesion*, commonly reflecting healed granulomatous disease (e.g., prior tuberculosis).
Cardiomegaly	Cardiomegaly	ThoracicAbnormality	Located at *Heart*; associated with *EnlargedCardiacSilhouettePattern*.
Consolidation	Consolidation	ThoracicAbnormality	Located in *Lung*; associated with *ConsolidationPattern* (dense parenchymal opacity).
ILD	ILD	ThoracicAbnormality	Located in *Lung*; associated with *InterstitialNodularPattern* and other interstitial changes consistent with diffuse parenchymal lung disease.
Infiltration	Infiltration	ThoracicAbnormality	Located in *Lung*; associated with *NonSpecificInfiltrativePattern*.
Lung opacity	LungOpacity	ThoracicAbnormality	Located in *Lung*; associated with broad *OpacityPattern* not otherwise specified.
Nodule/Mass	NoduleOrMassFinding	ThoracicAbnormality	Defined as *PulmonaryNodule* or *PulmonaryMass*; located in *Lung*; associated with *NodulePattern* or *MassPattern*.
Other lesion	OtherLesion	ThoracicAbnormality	Generic lesion class for abnormalities not covered by the other categories; location and pattern left unspecified.
Pleural effusion	PleuralEffusion	ThoracicAbnormality	Located in *PleuralSpace*, often involving *CostophrenicAngle*; associated with *EffusionPattern*.
Pleural thickening	PleuralThickening	ThoracicAbnormality	Located in *PleuralSpace*; associated with *PleuralThickeningPattern*.
Pneumothorax	Pneumothorax	ThoracicAbnormality	Located in *PleuralSpace*; associated with *PneumothoraxPattern* (air in the pleural cavity).
Pulmonary fibrosis	PulmonaryFibrosis	ThoracicAbnormality	Located in *Lung*, typically in *BasalZone*; associated with *FibroticPattern*.
No finding	NoFinding	NormalFinding	Represents absence of *ThoracicAbnormality*; used to describe *NormalCXRStudy*.

**Table 7:** Reproducibility configuration for OOD threshold calibration and evaluation. All detectors are computed using the same trained checkpoint and identical preprocessing for in-distribution (ID) and out-of-distribution (OOD) inputs.

Category	Setting
Random seed (OOD sampling)	42
ID calibration set	400 ID images from vinbig_val_10.csv
OOD benchmark set	400 ID (validation) vs. 400 non-thoracic bone radiographs
OOD list export	ood_bone_400.csv
Score export	ood_id_vs_bone_scores_400v400.csv
Image preprocessing	Resize to 1.15×384, centre-crop 384×384, ImageNet normalisation
Confidence score	$s_{\mathrm{conf}}=\max\left(\sigma \left(z\right)\right)$; OOD if $s_{\mathrm{conf}}<\tau_{\mathrm{conf}}$
Entropy score	$s_{\mathrm{ent}}=\frac{1}{C}\sum_{c=1}^{C}\left(-p_{c}\log p_{c}-\left(1-p_{c}\right)\log\left(1-p_{c}\right)\right)$, p=σ(z)
Energy score	$s_{\mathrm{energy}}=-\log\sum_{c=1}^{C}\exp\left(z_{c}\right)$, T=1.0
Mahalanobis distance	Diagonal Mahalanobis on fused features (D=1792), $\epsilon =10^{-3}$
Threshold calibration (ID)	$\tau_{\mathrm{conf}}={\mathrm{Quantile}}_{0.05}\left(s_{\mathrm{conf}}\right)$; $\tau_{\mathrm{ent}},\tau_{\mathrm{energy}},\tau_{\mathrm{Maha}}={\mathrm{Quantile}}_{0.95}\left(\cdot \right)$
OOD fusion rule	OR rule (OOD if any detector triggers)
Reported OOD metrics	AUROC, AUPRC, FPR95, and OR operating-point metrics

**Table 8:** Comparison to strong baselines and hybrid combinations on the VinBigData 20% hold-out test split.

Model	Test Macro-AUROC
VGG16 (CNN-only)	0.8694
ResNet50 (CNN-only)	0.8726
DenseNet121 (CNN-only)	0.8915
InceptionV3 (CNN-only)	0.8905
EfficientNet-B4 (CNN-only)	0.9002
ConvNeXtV (CNN-only)	0.9163
ViT-B/16 (Transformer-only)	0.9240
ResNet50 + ViT-B/16 (hybrid)	0.9250
EfficientNet-B4 + ViT-B/16 (hybrid)	0.9316
ConvNeXtV2-Base + ViT-B/16 + MXA (proposed)	0.9525

**Table 9:** Per-class performance on the 20% held-out test subset of VinBigData. AUROC is threshold-independent; we additionally report a 95% confidence interval (CI) computed via non-parametric bootstrap on the test set (*n* = 2000 resamples). Precision and F1-score are computed at fixed per-class decision thresholds fitted on the validation split and applied unchanged to the test split. Macro-average denotes the unweighted mean across classes; micro-average pools predictions across all label instances.

Class	AUROC	95% CI	Precision (%)	F1-Score (%)
Aortic enlargement	0.9825	[0.9711, 0.9890]	98.34	97.62
Atelectasis	0.9700	[0.9455, 0.9896]	87.80	92.31
Calcification	0.9151	[0.8935, 0.9451]	84.76	90.82
Cardiomegaly	0.9819	[0.9748, 0.9966]	93.42	95.98
Consolidation	0.9711	[0.9587, 0.9894]	89.61	93.24
ILD	0.9462	[0.9252, 0.9653]	91.46	94.34
Infiltration	0.9538	[0.9390, 0.9790]	93.08	95.65
Lung opacity	0.9380	[0.9348, 0.9644]	90.14	93.43
Nodule/Mass	0.9317	[0.9143, 0.9586]	86.02	91.17
Other lesion	0.9076	[0.8974, 0.9349]	80.22	87.60
Pleural effusion	0.9589	[0.9473, 0.9732]	91.36	94.37
Pleural thickening	0.9403	[0.9313, 0.9585]	89.58	93.48
Pneumothorax	0.9461	[0.9177, 0.9954]	94.44	91.89
Pulmonary fibrosis	0.9511	[0.9409, 0.9650]	91.12	94.64
No finding	0.9919	[0.9884, 0.9984]	97.28	98.42
Macro-average	0.9525	[0.9427, 0.9590]	90.58	93.66
Micro-average (pooled)	0.9777	[0.9736, 0.9791]	93.59	95.92

**Table 10:** Per-class AUROC comparison between hybrid variants on the VinBigData 20% hold-out test split.

Class	ResNet50 + ViT-B/16	EfficientNet-B4 + ViT-B/16	ConvNeXtV2-Base + ViT-B/16 + MXA (Proposed)
Aortic enlargement	0.9530	0.9620	0.9826
Atelectasis	0.9420	0.9530	0.9701
Calcification	0.8676	0.8946	0.9152
Cardiomegaly	0.9601	0.9632	0.9820
Consolidation	0.9302	0.9458	0.9712
ILD	0.9204	0.9246	0.9463
Infiltration	0.9234	0.9301	0.9538
Lung opacity	0.9269	0.9209	0.9380
Nodule/Mass	0.9187	0.9105	0.9318
Other lesion	0.8824	0.8934	0.9077
Pleural effusion	0.9236	0.9394	0.9590
Pleural thickening	0.9023	0.9180	0.9403
Pneumothorax	0.9425	0.9209	0.9461
Pulmonary fibrosis	0.9235	0.9360	0.9511
No finding	0.9592	0.9609	0.9920
Macro-average	0.9250	0.9316	0.9525

**Table 11:** Per-class CAM localisation on VinBigData at top-p=0.20. IoU denotes Mean IoU between the thresholded CAM region and the union of class-specific bounding boxes; Hit denotes any-overlap success rate. GC and GC++ refer to Grad-CAM and Grad-CAM++; pre/post refer to CAMs computed from the pre-MXA feature map ($f_{\mathrm{cnn}}$) and the post-MXA feature map ($a_{\mathrm{cnn}}$), respectively.

Class	IoU-GC (Pre)	Hit-GC (Pre)	IoU-GC (Post)	Hit-GC (Post)	IoU-GC++ (Pre)	Hit-GC++ (Pre)	IoU-GC++ (Post)	Hit-GC++ (Post)
Aortic enlargement	0.4291	98.0	0.4532	99.6	0.4121	98.5	0.4422	99.6
Atelectasis	0.4224	100.0	0.4423	100.0	0.4292	100.0	0.4326	100.0
Calcification	0.4111	100.0	0.4325	100.0	0.4125	100.0	0.4165	100.0
Cardiomegaly	0.4865	100.0	0.4997	100.0	0.4564	100.0	0.4887	100.0
Consolidation	0.3754	100.0	0.3955	100.0	0.3278	100.0	0.3955	100.0
ILD	0.3543	100.0	0.4347	100.0	0.3279	100.0	0.4627	100.0
Infiltration	0.3043	99.6	0.3991	100.0	0.2915	100.0	0.3811	100.0
Lung Opacity	0.3385	98.2	0.4138	100.0	0.3071	100.0	0.4038	100.0
Nodule/Mass	0.3519	99.2	0.4325	100.0	0.3119	100.0	0.4285	100.0
Other lesion	0.3066	100.0	0.3476	100.0	0.3066	100.0	0.3032	100.0
Pleural effusion	0.4175	100.0	0.4999	100.0	0.4075	100.0	0.4859	100.0
Pleural thickening	0.3716	100.0	0.3740	100.0	0.3616	100.0	0.3639	100.0
Pneumothorax	0.4270	100.0	0.4621	100.0	0.4103	100.0	0.4492	100.0
Pulmonary fibrosis	0.4400	99.6	0.4490	99.6	0.4310	99.6	0.4368	99.6
Macro-average	0.3883	99.62	0.4311	99.94	0.3710	99.86	0.4208	99.94

**Table 12:** Example of an obvious out-of-distribution (OOD) input (non-thoracic bone/foot radiograph). Thresholds correspond to in-distribution calibration on 400 VinBigData validation images. The combined decision uses an OR rule (OOD if any detector triggers).

Score	Measured Value	ID Threshold	OOD Flag
Max probability ($s_{\mathrm{conf}}$)	0.5410	0.7680	Yes
Mean Bernoulli entropy ($s_{\mathrm{ent}}$)	0.2616	0.3003	No
Energy score ($s_{\mathrm{energy}}$)	−0.9370	−1.5988	Yes
Mahalanobis ($s_{\mathrm{Maha}}$; diag)	71.6521	55.5607	Yes

**Table 13:** Per-detector OOD detection summary on the benchmark (400 ID VinBigData vs. 400 non-thoracic OOD). Thresholds (τ) are calibrated on 400 in-distribution validation images and then held fixed during evaluation. Metrics include AUROC, AUPRC, and FPR95 (ID false-positive rate at the threshold where OOD TPR = 0.95).

Detector	τ (Used)	AUROC	AUPRC	FPR95
Confidence (max p)	$\tau_{\mathrm{conf}}=0.7680$	0.9636	0.9241	0.1225
Entropy ($s_{\mathrm{ent}}$)	$\tau_{\mathrm{ent}}=0.3003$	0.9646	0.9365	0.0850
Energy ($s_{\mathrm{energy}}$)	$\tau_{\mathrm{energy}}=-1.5988$	0.9663	0.9032	0.1550
Mahalanobis ($s_{\mathrm{Maha}}$; diag)	$\tau_{\mathrm{Maha}}=55.5607$	0.9911	0.9824	0.0175

**Table 14:** Operating-point performance of the combined OR-based OOD decision using the fixed calibrated thresholds (OOD if any detector triggers), evaluated on 400 in-distribution and 400 out-of-distribution images.

Rule	TN	FP	FN	TP	Accuracy	Precision	Recall (TPR)	F1
Combined OR	386	14	0	400	0.9825	0.9662	1.0000	0.9828

**Table 15:** Per-class AUROC on the NIH ChestX-ray14 validation and test sets using the proposed ConvNeXtV2–ViT hybrid model. Class-specific thresholds (thr) were selected on the validation set and then kept fixed when evaluating the held-out test set.

Class	thr	VAL_AUROC	TEST_AUROC
Atelectasis	0.25	0.8824	0.8868
Cardiomegaly	0.25	0.954	0.9589
Consolidation	0.20	0.8735	0.8673
Edema	0.20	0.9332	0.9398
Effusion	0.40	0.9429	0.9414
Emphysema	0.35	0.9714	0.9694
Fibrosis	0.15	0.9158	0.9142
Hernia	0.65	0.9394	0.9711
Infiltration	0.25	0.7892	0.7690
Mass	0.35	0.9350	0.9302
Nodule	0.20	0.8912	0.8909
Pneumonia	0.10	0.8729	0.8604
Pleural Thickening	0.20	0.8918	0.8993
Pneumothorax	0.40	0.9495	0.9490
Macro-AUROC	–	0.9102	0.9106

**Table 16:** AUROC on the CheXpert validation set (frontal views only) for labels overlapping with the VinBigData task and with non-degenerate distributions.

Class	CheXpert VAL_AUROC
Atelectasis	0.8723
Cardiomegaly	0.7965
Consolidation	0.9284
Edema	0.8739
Effusion	0.9069
Pneumonia	0.8061
Pneumothorax	0.7568
Macro-AUROC	0.8487

## Data Availability

The chest radiographs used in this work were obtained from the publicly available VinBigData Chest X-ray Abnormalities Detection dataset, released for research use via a Kaggle competition. The dataset can be downloaded at https://www.kaggle.com/competitions/vinbigdata-chest-xray-abnormalities-detection (accessed on 14 October 2025).
